# Factors associated with maternal near miss in childbirth and the postpartum period: findings from the birth in Brazil National Survey, 2011–2012

**DOI:** 10.1186/s12978-016-0232-y

**Published:** 2016-10-17

**Authors:** Rosa Maria Soares Madeira Domingues, Marcos Augusto Bastos Dias, Arthur Orlando Corrêa Schilithz, Maria do Carmo Leal

**Affiliations:** 1 Instituto Nacional de Infectologia Evandro Chagas/Fundação Oswaldo Cruz, Av. Brasil, 4365 - Manguinhos, Rio de Janeiro, CEP 21040-360 Brasil; 2 Instituto Nacional de Saúde da Mulher, da Criança e do Adolescente Fernandes Figueira/Fundação Oswaldo Cruz, Av. Rui Barbosa, 716 - Flamengo, Rio de Janeiro, CEP 22250-020 Brasil; 3 Escola Nacional de Saúde Pública Sérgio Arouca/Fundação Oswaldo Cruz, Rua Leopoldo Bulhões, 1480 - Manguinhos, Rio de Janeiro, CEP 21041-210 Brasil

## Abstract

**Background:**

Maternal near-miss (MNM) audits are considered a useful approach to improving maternal healthcare. The aim of this study was to evaluate the factors associated with maternal near-miss cases in childbirth and the postpartum period in Brazil.

**Methods:**

The study is based on data from a nationwide hospital-based survey of 23,894 women conducted in 2011–2012. The data are from interviews with mothers during the postpartum period and from hospital medical files. Univariate and multivariable logistic regressions were performed to analyze factors associated with MNM, including estimation of crude and adjusted odds ratios and their respective 95 % confidence intervals (95 % CI).

**Results:**

The estimated incidence of MNM was 10.2/1,000 live births (95 % CI: 7.5–13.7). In the adjusted analyses, MNM was associated with the absence of antenatal care (OR: 4.65; 95 % CI: 1.51–14.31), search for two or more services before admission to delivery care (OR: 4.49; 95 % CI: 2.12–9.52), obstetric complications (OR: 9.29; 95 % CI: 6.69–12.90), and type of birth: elective C-section (OR: 2.54; 95 % CI: 1.67–3.88) and forceps (OR: 9.37; 95 % CI: 4.01–21.91). Social and demographic maternal characteristics were not associated with MNM, although women who self-reported as white and women with higher schooling had better access to antenatal and maternity care services.

**Conclusion:**

The high proportion of elective C-sections performed among women in better social and economic situations in Brazil is likely attenuating the benefits that could be realized from improved prenatal care and greater access to maternity services. Strategies for reducing the rate of MNM in Brazil should focus on: 1) increasing access to prenatal care and delivery care, particularly among women who are at greater social and economic risk and 2) reducing the rate of elective cesarean section, particularly among women who receive services at private maternity facilities, where C-section rates reach 90 % of births.

**Electronic supplementary material:**

The online version of this article (doi:10.1186/s12978-016-0232-y) contains supplementary material, which is available to authorized users.

## Background

Maternal mortality in Brazil showed a downward trend during the period 1990–2011, with an average annual decline in the maternal mortality ratio (MMR) of 3.7 %. However, the estimated MMR of 60.8 per 100,000 live births for 2011 [1] was still high compared with developed countries. These data contrast with observed improvements in other health indicators such as the expansion of primary healthcare [2] and virtually universal coverage of prenatal and hospital delivery care [3].

Possible explanations for this discrepancy are 1) poor quality of health services in Brazil, which has been reported in many studies evaluating the adequacy of antenatal care [4–8]; 2) the lack of integration between antenatal and maternity services, due to lack of services and/or service overcrowding, resulting in the search for one or more services during labor, for delivery care [9]; and 3) the increasing proportion of caesarean section, which might have serious effects on maternal health [10–13].

Maternal near miss (MNM) is defined by the World Health Organization (WHO) as “a woman who almost dies but survives the complication that occurred during pregnancy, childbirth or within 42 days after the end of pregnancy,” aligning with the definition of maternal death [14]. MNM audits are considered a useful approach to improving maternal healthcare [15] because cases of MNM are more frequent than maternal deaths and share the same problems and obstacles associated with care provided to women during pregnancy, childbirth, and the postpartum period [16–18].

The incidence of MNM by maternal characteristics has been estimated by Dias et al. [19] using data from the Birth in Brazil national survey (2011–2012) conducted among puerperal women who delivered in Brazilian hospitals. The aim of this study is to evaluate the factors associated with MNM in hospital delivery and the postpartum period using data from the Birth in Brazil survey.

## Methods

Almost 3 million births occur in Brazil each year with almost universal coverage of hospital delivery care [20]. Maternity care in Brazil is provided by a mix of public and private services. It has been estimated that, in 2011, 80 % of births were financed by the Public Health System and that 51.5 and 1.5 % of pregnant women had caesarean section or forceps delivery, respectively [21].

The “Birth in Brazil: national survey into labour and birth” was a hospital-based survey conducted between February 2011 and October 2012. The sample was selected in three stages. First, hospitals with more than 500 deliveries per year were stratified according to the five macro regions of the country (North, Northeast, South, Southeast, and Midwest), according to location (state capital or elsewhere in the state), and according to type of hospital service (public, mixed or private). This stratification followed the distribution of live births in 2007, according to the Information System on Live Births. A total of 266 hospitals were selected with probability proportional to the number of deliveries in each strata in 2007. In the second stage, the number of days needed to interview 90 puerperal women in each hospital—a minimum of 7 days—was selected using an inverse sampling method. In the third stage, eligible women were selected on each day of fieldwork. Sample losses because of refusal to participate or hospital discharge were replaced by selecting new puerperal women at the same hospital. Overall, interviews were conducted with 23,894 women. Additional information on the methodology used in the “Birth in Brazil” survey is available in Leal et al. [20] and Vasconcellos et al. [22].

All puerperal women who had a live birth in a hospital or a stillbirth in which the gestational age of the child was more than 22 weeks or the weight was greater than 500 g, as recorded in the medical file, were considered eligible for the survey. Miscarriages were excluded because the aim of the study was to evaluate the conditions of prenatal, birth and delivery care and the results of the care provided.

Interviews with the puerperal women were conducted during their hospital stay, at least 6 h after delivery, by the research team. Data from the medical files of the puerperal woman and newborn were obtained at the time of hospital discharge. In the event of prolonged hospitalization, data from medical files were obtained on the 42nd day of hospitalization for puerperal women or on the 28th day of life for newborns. Electronic forms developed specifically for the survey were used for the interview and for extraction of data from the medical files.

**Fig. 1:**
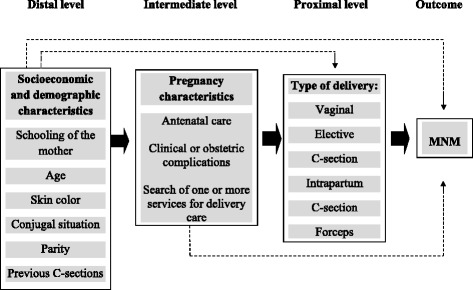
Theoretical model of the determinants of maternal near miss (MNM)

Univariate and multivariable statistical analyses of MNM-associated factors were carried out using non-conditional logistic regression, following the hierarchical model [23] presented in Fig. 1. At the distal level, socioeconomic and demographic variables were included: age (12–19 years, 20–34 years, ≥35 years); schooling level (≤7 years, 8–10 years, 11–14 years, ≥15 years of school attendance); self-reported skin color (white, black, mixed, East Asian, indigenous); conjugal situation (living with partner or not); parity (primiparous or not), and number of previous C-sections (none, one, two or more). At the intermediate level, pregnancy-related variables included: antenatal care (at least one prenatal consultation); clinical or obstetrical complications (yes or no); and number of maternity services searched before hospital admission (none, one, two or more services). At the proximal level, the only variable was type of delivery (elective C-section, intrapartum C-section, vaginal, and forceps). The outcome was the incidence of MNM.

Clinical or obstetric complications were defined as conditions that constitute possible indications for C-section and also be potentially associated with increased maternal morbidity. According to hospital file records, women who presented with one of the following criteria were considered to present clinical or obstetric complications: hypertensive disorders (chronic hypertension, pre-eclampsia, eclampsia, and HELPP syndrome), diabetes, placenta previa, placental abruption, HIV infection, and other maternal infection at hospital admission.

C-sections were classified based on information recorded in hospital files. C-sections were defined as elective if: a) the woman had no labor or b) the woman had spontaneous or induced labor but underwent cesarean section when uterine dilatation was less than 4 cm [24]. All other caesareans were classified as intrapartum C-sections, no matter the duration of labor or the indication of the caesarean.

Cases of MNM were identified according to WHO criteria [14], using information contained in hospital patient records. All cases identified were reviewed by two specialists independently, with a view to detecting possible inconsistencies in extraction of data from patient records or completion of patient records. Disagreements were resolved by consensus.

A univariate analysis estimated the unadjusted odds ratio (OR) and 95 % confidence interval (95 % CI). The first multivariable model included all the distal variables. Variables from the first model with a significance level of <0.20 were included in the second model, along with all the intermediate level variables. The third model included distal and intermediate level variables with a significance level of <0.20, along with the proximal variables. All variables with a significance level of <0.05 were retained in the final multivariable model. The results from the final multivariable model were expressed as adjusted odds ratios with their corresponding 95 % confidence intervals (95 % CI).

To analyze the association of socioeconomic and demographic variables with pregnancy variables, the chi-squared statistical test was used to verify differences between proportions with a significance level of <0.05.

The complex sampling design was taken into consideration in all the statistical analyses. Weighting of the data was calculated according to the inverse of the probability of inclusion of each puerperal woman in the sample. To ensure that the distribution of the puerperal women interviewed was similar to that observed among the births in the population sampled in 2011, a calibration procedure was used in each selection stratum [22]. For the univariate and multivariable logistic regressions, women who self-reported as East Asian or indigenous were excluded because they accounted for a very small proportion of the sample (1.5 %). The analyses were performed using IBM SPSS Statistics for Windows, Version 19.0 (IBM Corp., Armonk, NY, USA).

This study was approved by the Research Ethics Committee of ENSP/Fiocruz, under report no. 92/2010. Care was taken to ensure privacy and confidentiality regarding the information collected from the women. Informed consent was obtained before the interview through the use of an informed consent statement.

## Results

The mean age of the 23,894 puerperal women interviewed was 25.7 years; the median age was 25 years, with 19.1 % between the age of 12 and 19 years. Half of the respondents had attended school for up to 10 years and the majority self-reported their skin color as mixed (56.1 %), whereas East Asians and indigenous women accounted for 1.1 and 0.4 % of the sample, respectively. More than 80 % of the women lived with a partner, 46.9 % were primiparous, and 16.2 % had had previous C-sections. Nearly 99 % of the puerperal women had had at least one prenatal consultation, with a mean of 7.17 and median of 7 visits; 16.2 % searched one or more maternities before admission; and 19.2 % had at least one clinical or obstetric complication during pregnancy, childbirth, or the postpartum period. Almost half of the women had had a vaginal birth, whereas 43.7 % had an elective C-section, 8.2 % had an intrapartum C-section, and 1.5 % had a forceps operative vaginal delivery (Table 1).

**Table 1:** Percent distribution of puerperal women (*n* = 23,894) included in a study of hospitalization for childbirth care by maternal characteristics, Brazil, 2011–2012

Characteristic	Number	Percent
Maternal age (years)		
12–19	4571	19.1
20–34	16,807	70.4
35 and over	2509	10.5
Schooling level (years)		
15 or more	2107	8.9
11–14	9263	39.0
8–10	6086	25.6
0–7	6322	26.6
Skin color		
White	8077	33.8
Mixed	13,404	56.1
Black	2051	8.6
East Asian	257	1.1
Indigenous	99	0.4
Conjugal situation		
With partner	19,440	81.4
Without partner	4432	18.6
Number of previous births		
0	11,208	46.9
1	7014	29.4
2–3	4501	18.8
4 or more	1171	4.9
Previous C-section		
0	18,779	78.6
1	3905	16.3
2 or more	1211	5.1
Number of Antenatal care visits		
0	286	1.2
1–3	2123	9.1
4–5	4110	17.6
6 or more	16,898	72.1
Number of services before admission to childbirth care		
0	20,005	83.8
1	3302	13.8
2 or more	569	2.4
Clinical or obstetric complications		
No	19,264	80.6
Yes	4630	19.4
Type of clinical or obstetric complications^a^		
Hypertensive disorders	2656	11.1
Diabetes mellitus	1968	8.2
Placental abruption	310	1.3
Placenta previa	116	0.5
Maternal infections	83	0.3
HIV infection	96	0.4
Type of delivery		
Vaginal	11,152	46.7
Elective C-section	10,436	43.7
Intrapartum C-section	1959	8.2
Forceps	347	1.5

Totals for these variables vary because of missing values

^a^Only women with clinical or obstetric complications (*n* = 4630)

The incidence of MNM was 10.2 per 1000 live births (95 % CI: 7.5–13.7 per 1000). In the unadjusted analysis, greater incidence of MNM was observed among women aged 35 years or over, women who had two or more previous C-sections, women who searched for two or more services before hospital admission, women who had clinical or obstetric complications during pregnancy, and women who had an elective C-section or a forceps delivery. Women without antenatal care had MNM incidence of 27.97 per 1000 live births, with odds of 2.90 but borderline statistical significance (95 % CI: 0.94–8.92; *p* value = 0.064). Parity also had borderline statistical significance (*p* value = 0.061) but with a lower OR (1.3) and a narrow confidence interval (95 % CI: 0.99–1.79). No differences regarding schooling level, self-reported skin color or conjugal situation were observed (Table 2).

**Table 2:** Incidence of maternal near miss (MNM), odds ratio (OR), 95 % confidence interval, and probability of MNM among puerperal women (*n* = 23,894) included in the study, according to maternal characteristics, Brazil, 2011–2012

Characteristic	MNM incidence ^a^	OR^b^	95 % CI^c^	p
Maternal age (years)				
12–19	10.06	1.07	0,72–1.60	
20–34	9.40	1	1	0.081
35 and over	15.54	1.65	1.07–2.55	
Schooling level (years)				
15 or more	6.17	1		
11–14	10.04	1.61	0.75–3.45	
8–10	11.18	1.81	0.84–3.92	0.494
0–7	10.91	1.75	0.72–4.22	
Skin color^d^				
White	9.29	1		
Mixed	10.67	1.16	0.79–1.70	0.746
Black	9.75	1.08	0.45–2.56	
Conjugal situation				
With partner	10.02	1		
Without partner	10.83	1.08	0.77–1.52	0.651
Primipara				
No	8.82	1		
Yes	11.68	1.33	0.99–1.79	0.061
Previous C-section				
0	9.58	1		
1	10.84	1.13	0.75–1.72	
2 or more	17.05	1.79	1.09–2.97	0.035
Antenatal care				
Yes	9.87	1		
No	27.97	2.90	0.94–8.92	0.064
Number of services before admission to delivery care				
0	8.85	1		
1	13.93	1.59	0.89–2.83	
2 or more	35.15	4.10	1.97–8.52	0.001
Clinical or obstetric complications^e^				
No	3.63	1		
Yes	37.15	10.55	7.61–14.63	<0.001
Type of delivery				
Vaginal	4.39	1		
Elective C-section	16.00	3.70	2.47–5.55	
Intrapartum C-section	6.64	1.48	0.76–2.88	
Forceps	40.35	9.64	4.24–21.91	<0.001
TOTAL	10.16	--	7.14–13.18	--

Totals for these variables vary because of missing values

^a^Incidence of maternal near miss per 1000 live births.^b^OR = odds ratio; ^c^ CI = confidence interval; ^d^ pregnant women who self-reported with East Asian or indigenous skin color were excluded from this analysis; ^e^ women who presented with one of the following criteria were considered to present clinical or obstetric complications: hypertensive disorders, diabetes, placenta previa, placental abruption, HIV infection and other maternal infections

The results from the adjusted analysis are presented at Table 3. In the first model (Model 1) women’s age, schooling level, parity, and number of previous C-sections had a significance level of <0.20 and were included in the second model (Model 2). In the second model, schooling level, parity, previous C-sections, and all the intermediate variables had a significance level of <0.20 and were included in the third model (Model 3). In the third model, only the intermediate variables (antenatal care, search for two or more services, clinical or obstetric complications) and proximal variables (type of delivery) had a significance level of <0.05 and were kept in the final model (Final Model).

**Table 3:** Multivariable logistic regression on the maternal characteristics associated with incidence of maternal near miss during hospitalization for childbirth care (*n* = 23,894), Brazil, 2011–2012

Characteristic	Model 1^a^			Model 2^b^			Model 3^c^			Final Model^d^		
	OR^e^	95 % CI^f^	p	OR^e^	95 % CI^f^	p	OR^e^	95 % CI^f^	p	OR^e^	95 % CI^f^	p
Maternal age (years)												
12–19	0.71	0.48–1.06		0.96	0.60–1.52							
20–34	1			1								
35 and over	1.93	1.25–2.97	0.001	1.37	0.87–2.17	0.358						
Schooling level (years)												
15 or more	1			1			1					
11–14	1.96	0.91–4.24		1.65	0.81–3.39		1.78	0.85–3.73				
8–10	2.49	1.15–5.37		2.17	1.04–4.54		2.48	1.15–5.33				
0–7	2.66	1.04–6.78	0.106	2.07	0.84–5.08	0.185	2.51	0.99–6.36	0.075			
Skin color^g^												
White	1											
Mixed	1.15	0.76–1.75										
Black	1.05	0.43–2.56	0.777									
Conjugal situation												
With partner	1											
Without partner	0.97	0.67–1.42	0.893									
Primipara												
No	1			1			1					
Yes	2.40	1.53–3.77	<0.001	2.02	1.26–3.25	0.004	1.45	0.90–2.34	0.131			
Previous C-section												
0	1			1			1					
1	1.89	1.06–3.38		1.61	0.91–2.88		1.07	0.62–1.86				
2 or more	2.64	1.62–4.30	<0.001	2.13	1.31–3.45	0.003	1.30	0.74–2.29	0.638			
Antenatal care												
Yes				1			1			1		
No				4.15	1.34–12.81	0.014	4.44	1.37–14.37	0.013	4.65	1,51–14.31	0007
Number of services before admission to delivery care												
0				1			1			1		
1				1.50	0.84–2.68		1.57	0.89–2.75		1.69	0.98–2.92	
2 or more				3.88	1.84–8.18	0.002	4.05	1.92–8.54	<0.001	4.49	2.12–9.52	<0.001
Clinical or obstetric complications^h^												
No				1			1			1		
Yes				10.28	7.51–14.06	<0.001	9.26	6.66–12.87	<0.001	9.29	6.69–12.90	<0.001
Type of delivery												
Vaginal							1			1		
Elective C-section							2.67	1.70–4.18		2.54	1.67–3.88	
Intrapartum C-section							1.02	0.53–1.94		1.05	0.54–2.03	
Forceps							9.08	3.92–21.06	<0.001	9.37	4.01–21.91	<0.001

^a^Model 1 = analyses were adjusted for age, schooling level, self-reported skin color, conjugal situation, parity and number of previous C-sections;^b^Model 2 = analyses were adjusted for age, schooling level, parity, number of previous C-sections, antenatal care, diagnoses of clinical or obstetric emergencies, number of services searched before admission to delivery care; ^c^Model 3 = analyses were adjusted for schooling level, parity, number of previous C-sections, antenatal care, diagnoses of clinical or obstetric emergencies, number of services searched before admission to delivery care and type of delivery; ^d^Final Model = analyses were adjusted for antenatal care, diagnoses of clinical or obstetric emergencies, number of services searched before admission to delivery care and type of delivery; ^e^OR = odds ratio; ^f^CI = confidence interval; ^g^Pregnant women with East Asian or indigenous skin color were excluded from this analysis; ^h^Women who presented with one of the following criteria were considered to present clinical or obstetric complications: hypertensive disorders, diabetes, placenta previa, placental abruption, HIV infection and other maternal infections

The odds of MNM among women who received no antenatal care was 4.65 higher (95 % CI: 1.51–14.31) than among women who had at least one consultation. Women who reported the search of two or more maternities before admission had an odds of MNM incidence four times higher than those who were admitted at the first childbirth care service (OR: 4.49; 95 % CI: 2.12–9.52). Women with clinical or obstetric complications had the highest odds of MNM in the unadjusted analysis and an odds of 9.29 (95 % CI: 6.69–12.90) after adjustment for other variables. Women with elective C-section had odds of MNM of 2.54 (95 % CI: 1.67–3.88) after adjustment for other variables, whereas women with a forceps delivery had the highest odds of MNM incidence (OR: 9.37; 95 % CI: 4.01–21.91).

Table 4 shows the association of selected social and demographic maternal characteristics with antenatal care, childbirth services use, and clinical or obstetrical complications. Women aged 35 years or over presented with more clinical or obstetrical complications and had more elective C-sections, whereas women younger than 20 years reported searching for more than one service for childbirth care. No differences related to mother’s age were observed for antenatal care. Women with less than 8 years of schooling reported less antenatal care. A gradient was observed for the proportion of elective C-sections and the number of years of schooling: the higher the level of schooling, the higher the proportion of elective C-section. Inversely, the proportion of women who reported the search of one or more childbirth care services before hospital admission increased as the number of years of schooling decreased. Women who self-reported as black or mixed skin color had less antenatal care, reported searching for more than one service for hospital admission, and had fewer elective C-sections; at the same time, black women had more clinical and obstetrical complications. A similar pattern was observed among women without a partner; they had less access to antenatal and maternity care services and had fewer elective C-sections. Women with previous C-sections had more complications during pregnancy, but had greater access to maternity care and had almost three times as many elective C-sections as women with no previous caesarean sections.

**Table 4:** Use of antenatal care, search for childbirth services, clinical and obstetrical complications, and elective C-section, according to maternal characteristics, Brazil, 2011–2012

Characteristic	Antenatal care	p^a^	Search of one or more services before admission	p^a^	Clinical or obstetric complications	p^a^	Elective C-section	p^a^
Maternal age (years)								
12–19	98.5		21.3		12.9		26.8	
20–34	98.8		15.4		19.2		46.0	
35 and over	98.9	0.745	12.5	<0.001	32.2	<0.001	59.0	<0.001
Schooling level (years)								
15 or more	100		5.9		20.5		79.5	
11–14	99.5		14.0		20.8		50.9	
8–10	98.9		18.5		17.8		35.4	
0–7	97.3	<0.001	20.7	<0.001	18.5	0.009	29.0	<0.001
Skin color^b^								
White	99.3		12.2		20.3		53.1	
Mixed	98.6		18.1		18.5		39.5	
Black	98.5	0.009	19.6	<0.001	22.4	0.004	35.0	<0.001
Conjugal situation								
With partner	99.2		15.8		19.8		45.3	
Without partner	97.2	<0.001	18.1	0.041	17.5	0.035	36.4	<0.001
Primipara								
No	98.2		14.8		19.8		41.5	
Yes	99.5	<0.001	17.8	<0.001	18.9	0.264	46.1	<0.001
Previous C-section								
0	98.8		17.1		18.2		35.0	
1	99.0		13.3		23.2		71.5	
2 or more	98.8	0.200	11.3	<0.001	25.6	<0.001	89.2	<0.001

^a^Chi-squared statistic test; ^b^Pregnant women who self-reported with East Asian or indigenous skin color were excluded from this analysis

## Discussion

This study estimated an incidence of MNM of 10.2/1,000 live births during hospitalization for childbirth care. Similar rates were found in studies conducted by Souza et al. [25, 26] and Morse et al. [27], that also used WHO MNM criteria. Galvão et al. [28], in a study conducted in two public maternity services in the state of Sergipe/Brazil, reported a lower MNM rate of 4.7/1,000 live births. Comparison of the MNM results from our analysis with the results produced by other studies [29] is limited because we used the WHO MNM criteria in our study while other studies used criteria adopted previously that included different criteria for the definition of MNM [30].

MNM was associated with the absence of antenatal care (ANC), the search of two or more services before admission to delivery care, clinical or obstetric complications, and type of birth (elective cesarean section and forceps).

Studies have demonstrated the effectiveness of different practices routinely performed in ANC to prevent maternal and perinatal morbidity and mortality [31, 32]. Observational studies have also demonstrated the benefits of such assistance, correlating more consultations with more favorable outcomes [33–37], although there is no consensus on the optimal number of prenatal consultations for pregnant women [38, 39]. In this study, women were classified as attending antenatal care if they reported at least one consultation during pregnancy. Although having only one antenatal care consultation is not the recommended practice, many procedures can be offered in one visit like test and treatment for syphilis and HIV; diagnosis of clinical complications like hypertensive disorders; counselling about risk factors; referral to high risk pregnancy services; and linkage to maternity service. Nonetheless, more than 70 % of the women had the appropriate number of consultations—a minimum of six consultations is recommended for a term pregnancy by the Brazilian Ministry of Health. In this study, the absence of antenatal care was associated with less years of schooling, with black or mixed skin colour, with not living with a partner and with having previous births. Results from the study “Birth in Brazil” published elsewhere [9] demonstrated that the absence of antenatal care was also associated with living in the less developed North region, with previous negative pregnancy outcomes and with dissatisfaction with the current pregnancy. The most frequent reported reasons for not attending prenatal care were access barriers and personal problems (43.2 and 40.6 %, respectively). These results suggest that in Brazil, the absence of at least one antenatal care visit, in a context of almost universal antenatal care coverage, is associated with social vulnerabilities and barriers to access to health services that can enhance the risk of adverse outcomes.

The search for two or more services for hospital admission for birth care causes a delay in access to adequate care on entering the healthcare facility, with adverse effects such as complications and even death [40, 41]. Pacagnella et al. [42], in a national multi-center cross-sectional study in 27 Brazilian hospitals, demonstrated that the occurrence of any delay was associated with increasing severity of maternal outcome: 52 % in potentially life-threatening conditions, 68.4 % in maternal near-miss, and 84.1 % in maternal death. Overall, any type of delay was observed in 53.8 % of cases and 34.6 % of delays were related to health service accessibility. Although Brazilian laws [43] and protocols [44] regulate the linkage of pregnant women to the maternity of reference for childbirth care, in this study 16 % of women reported the search of one or more services for delivery care. This finding reveals a lack of integration between antenatal and childbirth care. The search of one or more services was associated with lower age, with less years of schooling, with black or mixed skin colour, with previous births and with not living with a partner. These are characteristics of women that use public maternity services. Women of better social and economic conditions, cared for in private services, usually do not have to search for more than one service, as usually the same professional provides antenatal and intrapartum care.

Clinical and obstetric complications are the main causes of maternal mortality and severe morbidity, and reinforce the importance of adequate care for women with high-risk pregnancies. The criteria of clinical or obstetrical complications adopted in this study included pathologies that could result in potentially life-threatening conditions, such as hypertensive disorders, hemorrhage, sepsis, or severe systemic infection [45]. HIV infection was included because it has affected the decline of maternal mortality in some countries [46] and is a common indication of C-section.

Both elective C-sections and forceps were associated with MNM. C-sections have been described as a cause of maternal death [12, 13] and near miss [13] and are associated with increased risk of blood transfusion [11, 12], bleeding complications [47], infections [47, 48], hysterectomies [11, 12, 48, 49], admission to intensive care unit [11], hospital stays of more than 7 days [11], and antibiotic treatment after delivery [11]. In this study, after adjustment for pregnancy complications, social and demographic variables, and antenatal care, elective C-sections more than doubled the odds of MNM. Similar results were described by Villar et al. [11] in a study of 410 health facilities in 24 areas in eight randomly selected Latin American countries. Women who had a forceps vaginal delivery had the highest odds of MNM after adjustment for other variables. However, forceps delivery accounted for only 1.5 % of childbirths and very few cases of MNM can actually be attributed to the use of forceps. Although elective cesarean section has lower odds than forceps delivery, the effect on MNM is greater because elective C-section accounts for more than 40 % of deliveries in Brazil.

The rate of caesarean section has been increasing in Brazil since the mid-1990s and it has been the main type of delivery in the country since 2009 [50]. Higher rates have been observed in older women, women with more education, primiparous women, women who receive prenatal care in the private sector, and women living in the South, Southeast, and Midwest [51], being determined in many cases by no clinical factors [52–55]. The rates of C-section in private services are typically between 80 and 90 %, with 80 % of caesareans performed before labor begins [21]. It should be noted that a recent study [56] corroborates the statement that a population-level cesarean section rate above 10–15 % is hardly justified from the medical perspective. The WHO estimates that more than 1 million unnecessary caesareans are performed in Brazil every year [57].

Previous C-sections were not associated with MNM, after adjusting for other variables in the model, while the results from other studies are controversial [23, 58]. In this study, more than 70 % of pregnant women with a previous C-section were submitted to an elective C-section. The findings suggest that the risk of MNM associated with a previous C-section is mitigated by the elective C-section and, in the current pregnancy, it is the type of delivery that accounts for most of the associated risk with MNM cases because it is closer to the outcome variable.

Age has been reported as a risk factor for MNM [59]. In this study, women aged 35 years and older, as well as those who self-reported as black skin color, were more likely to have clinical or obstetric complications; however, after adjustment for other variables, socioeconomic and demographic characteristics were no longer associated with MNM. A possible explanation for the reduced importance of social and economic characteristics in the study findings can be seen in an examination of two groups. First, women who self-reported as white and who had 8 or more years of schooling reported greater access to ANC and were more often admitted to the first maternity service searched for delivery care, but had higher proportions of elective C-section. Second, women who self-reported as black or mixed and who had less than 8 years of schooling reported less ANC care and searched for more services before admission for delivery care, but had lower rates of elective C-section. These results suggest that the high proportion of elective C-section is attenuating the maternal health benefits that result from antenatal care and greater access to maternity services—typically associated with women living in better social and economic conditions—thus equalizing the risk of MNM among these women with the risk of MNM among women living in poor social and economic conditions who, theoretically, are at higher risk for negative outcomes.

Souza et al. [25] reported a protective effect of low maternal education against the occurrence of MNM in the 2005 WHO global survey on maternal and perinatal health. Caesarean section has been reported to increase maternal morbidity in Latin America [10, 11] where women with lower education are known to undergo fewer C-sections. The authors suggest that the increase in C-section rates may be linked to iatrogenic maternal morbidity and maternal deaths [25].

Similar results have been described for neonatal mortality in the South region of Brazil [60]. An increased rate of preterm births, observed during the period 1982–2004, seemed to result largely from C-sections or inductions. Although newborn care had improved, and gestational-age-specific mortality rates had fallen, neonatal mortality rates remained stable since 1990, probably because of the increase in preterm births. The authors conclude that the excessive use of interventions during pregnancy and childbirth might have offset the gains resulting from improved maternal health and newborn survival.

This study was conducted in institutions where more than 500 deliveries take place each year. It is likely that pregnant women who have a planned or unplanned out of hospital delivery or who deliver in a smaller hospital would have different risks for MNM. Nevertheless, given that more than 99 % of deliveries in Brazil take place in hospitals, and approximately 80 % are in larger hospitals [20], significant changes to the results presented would not be expected.

Miscarriages were not included in this study, which may have affected the estimation of MNM incidence, because miscarriages are a known cause of MNM and death. One of the objectives of this study was to determine the association of MNM with type of delivery; the exclusion of miscarriages does not affect this analysis. It is possible, however, that other factors are associated with MNM in cases of abortion.

MNM cases were identified using information available in hospital patient records. It is possible that failures in recording medical file data may have led to an underestimation of MNM cases. Because this is a non-differential misclassification with respect to the factors studied, it is expected that there has been attenuation of the magnitude of the observed associations.

Finally, the WHO criteria adopted for classification of MNM cases may hamper comparison with other studies that have used different criteria. It is likely, however, that analysis of factors associated with MNM cases are not impaired by the criteria used, although one study has suggested that when management criteria are used in isolation [61] there is a tendency to include less severe cases, which might limit comparison of risk factors.

## Conclusions

The results of this study demonstrate that the absence of ANC, complications during pregnancy, search for two or more services for childbirth care, and type of delivery (elective cesarean section and forceps) are associated with MNM cases during hospitalization for delivery care in Brazil.

Two strategies seem necessary to reduce the rate of MNM. For women with greater social vulnerability, investment in access to ANC and maternity services are necessary to facilitate the early identification of pregnancy, provide adequate ANC, and ensure the linkage of pregnant women to maternity care where labor and delivery will take place. For women in better social and economic conditions, served in large part by private maternity services—where rates of caesarean section reach 90 % of births—strategies to reduce the rate of elective cesarean section are crucial.

## Additional files

Additional file 1: Translated article. (DOCX 113 kb)
Additional file 2: Reviewer reports. (PDF 251 kb)

## Abbreviations

ANC: Antenatal care
MMR: Maternal mortality ratio
MNM: Maternal near miss
WHO: World Health Organization
