# Supplementary material for: Factors associated with maternal near miss in childbirth and the postpartum period: findings from the birth in Brazil National Survey, 2011–2012
Source: Reprod Health. 2016 Oct 17;13(Suppl 3):115. doi: 10.1186/s12978-016-0232-y (PMC5073804; doi:10.1186/s12978-016-0232-y)
Supplement: Additional file 1: — Translated article. (DOCX 113 kb) [file 12978_2016_232_MOESM1_ESM.docx]

**Fatores associados ao *near miss* materno durante o parto e o pós-parto: resultados do inquérito nacional “Nascer no Brasil”, 2011-2012**

Rosa Maria Soares Madeira Domingues^1*^, Marcos Augusto Bastos Dias^2^, Arthur Orlando Corrêa Schilithz^3^ e Maria do Carmo Leal^3^

* Correspondência: Rosa Maria Soares Madeira Domingues. rosa.domingues@ini.fiocruz.br

^1^ Instituto Nacional de Infectologia Evandro Chagas/Fundação Oswaldo Cruz, Av. Brasil, 4365 - Manguinhos, Rio de Janeiro, Brasil. CEP 21040-360

^2^ Instituto Nacional de Saúde da Mulher, da Criança e do Adolescente Fernandes Figueira/Fundação Oswaldo Cruz, Av. Rui Barbosa, 716 - Flamengo, Rio de Janeiro, Brasil. CEP 22250-020

^3^ Escola Nacional de Saúde Pública Sérgio Arouca/Fundação Oswaldo Cruz, Rua Leopoldo Bulhões, 1480 - Manguinhos, Rio de Janeiro, Brasil. CEP 21041-210

**Resumo**

**Introdução**

A auditoria de casos de *Near Miss* Materno (NMM) é considerada uma abordagem útil para melhorar a qualidade da assistência à saúde materna. O objetivo deste estudo foi avaliar os fatores associados aos casos de *near-miss* materno no parto e no período pós-parto no Brasil.

**Métodos**

Estudo baseado em dados de uma pesquisa nacional de base hospitalar com 23.894 mulheres realizada em 2011-2012. Os dados são provenientes de entrevistas com as mulheres durante o período do pós-parto e de prontuários hospitalares. Regressão logística uni e multivariada foram realizadas para analisar os fatores associados ao NMM, incluindo estimativa da razão de chance bruta e ajustada e seus respectivos intervalos de confiança de 95% (IC 95%).

**Resultados**

Incidência estimada de NMM de 10,2 / 1.000 nascidos vivos (IC 95%: 7,5-13,7). Nas análises ajustadas, NMM esteve associado à ausência de assistência pré-natal (RC: 4,65; IC 95%: 1,51-14,31), procura de dois ou mais serviços para assistência ao parto (RC: 4,49; IC 95%: 2.12- 9.52), complicações obstétricas (RC: 9,29; IC 95%: 6,69-12,90), e tipo de nascimento: cesariana eletiva (RC: 2,54; IC 95%: 1,67-3,88) e forceps (RC: 9,37; 95% IC: 4,01-21,91). Características sociais e demográficas maternas não mostraram associação com NMM, embora as mulheres que se auto-declararam como brancas e as mulheres com maior escolaridade tenham apresentado melhor acesso aos serviços de pré-natal e ao parto.

**Conclusão**

A alta proporção de cesarianas eletivas realizadas entre as mulheres em melhores situações sociais e econômicas no Brasil está provavelmente atenuando os benefícios que poderiam advir de uma melhor assistência pré-natal e maior acesso a serviços de atenção ao parto. Estratégias para redução da taxa de NMM no Brasil devem concentrar-se: 1) no aumento do acesso à assistência pré-natal e ao parto, particularmente para as mulheres de maior risco social e econômico e 2) na redução da taxa de cesariana eletiva, particularmente entre as mulheres atendidas em serviços privados, onde as taxas de cesariana alcançam 90% dos nascimentos.

**Introdução**

A mortalidade materna no Brasil apresentou uma tendência descendente durante o período 1990-2011, com uma diminuição média anual da taxa de mortalidade materna (RMM) de 3,7%. No entanto, a RMM estimada de 60,8 por 100.000 nascidos vivos em 2011 [1] foi ainda elevada quando comparada às taxas observadas em países desenvolvidos. Esses dados contrastam com as melhorias observadas em outros indicadores de saúde, como a expansão dos cuidados de saúde primários [2] e a cobertura praticamente universal de assistência pré-natal e ao parto hospitalar [3].

Possíveis explicações para esta discrepância são: 1) a má qualidade dos serviços de saúde no Brasil, que tem sido relatados em muitos estudos avaliando a adequação da assistência pré-natal [4] - [8]; 2) a falta de integração entre os serviços de pré-natal e de atenção ao parto, devido à falta de serviços e / ou a superlotação dos mesmos, resultando na busca de um ou mais serviços para assistência ao parto durante o trabalho de parto [9]; e 3) o aumento da proporção de cesarianas, que podem ter graves repercussões sobre a saúde materna [10] - [13].

O *Near Miss* Materno (NMM) é definido pela Organização Mundial de Saúde (OMS) como "uma mulher que quase morreu, mas que sobreviveu a complicação que ocorreu durante a gravidez, o parto ou no prazo de 42 dias após o término da gravidez", alinhando-se com a definição de morte materna [14]. A auditoria de casos de NMM é considerada uma abordagem útil para melhoria da saúde materna [15] porque casos de NMM são mais frequentes do que casos de morte materna e compartilham os mesmos determinantes e barreiras relacionados com a assistência prestada às mulheres durante a gravidez, o parto e o período pós-parto [16] - [18].

A incidência de NMM segundo características maternas foi estimada por Dias et al. [19] utilizando dados da pesquisa nacional “Nascer no Brasil” (2011-2012) realizada com puérperas que tiveram parto em hospitais brasileiros. O objetivo deste estudo é avaliar os fatores associados ao NMM durante o parto e o pós-parto hospitalar utilizando dados da pesquisa “Nascer no Brasil”.

**Métodos**

Aproximadamente 3 milhões de nascimentos ocorrem no Brasil a cada ano com uma cobertura quase universal da assistência ao parto hospitalar [20]. A assistência ao parto no Brasil é prestada por um mix de serviços públicos e privados. Estima-se que, em 2011, 80% dos nascimentos foram financiados pelo Sistema Único de Saúde e que 51,5% e 1,5% das gestantes apresentaram cesariana ou parto a fórceps, respectivamente [21].

O estudo “Nascer no Brasil”, pesquisa nacional de base hospitalar, foi realizado entre Fevereiro de 2011 e Outubro de 2012. A amostra foi selecionada em três etapas. Na primeira etapa, hospitais com mais de 500 partos por ano foram estratificados de acordo com as cinco macrorregiões do país (Norte, Nordeste, Sul, Sudeste e Centro-Oeste), de acordo com a localização (capital do estado ou interior), e de acordo com tipo de serviço hospitalar (público, misto ou privado). Esta estratificação seguiu a distribuição de nascidos vivos em 2007, de acordo com o Sistema de Informação sobre Nascidos Vivos. Um total de 266 hospitais foram selecionados com probabilidade proporcional ao número de partos em cada estrato em 2007. Na segunda etapa, o número de dias necessários para entrevistar 90 puérperas em cada hospital - um mínimo de 7 dias - foi selecionado usando um método de amostragem inversa. Na terceira etapa, as mulheres elegíveis foram seleccionados em cada dia de trabalho de campo. Perdas por recusa em participar ou por alta hospitalar antes da realização da entrevista foram repostas pela inclusão de novas puérperas selecionadas no mesmo hospital. No total, foram realizadas entrevistas com 23.894 mulheres. Informações adicionais sobre a metodologia utilizada na pesquisa "Nascer no Brasil" podem ser encontradas em Leal et al. [20] e Vasconcellos et al. [22].

Todas as puérperas que tiveram um parto hospitalar com nascimento vivo, ou um natimorto em que a idade gestacional era maior do que 22 semanas ou o peso maior do que 500 g, conforme registrado no prontuário hospitalar, foram consideradas elegíveis para o estudo. Abortos foram excluídos porque o objetivo principal do estudo era avaliar a assistência pré-natal e ao parto prestada e e os resultados dessa assistência.

Entrevistas com as puérperas foram realizadas pela equipe da pesquisa durante a internação hospitalar, num intervalo mínimo de 6 horas após o parto. Dados do prontuário da puérpera e do recém-nato foram extraídos no momento da alta hospitalar. Em caso de hospitalização prolongada, os dados dos prontuários foram obtidos no 42º dia de hospitalização da puérpera ou no 28º dia de vida do recém-nascido. Formulários eletrônicos desenvolvidos especificamente para a pesquisa foram utilizados para a entrevista e para a extração de dados dos prontuários hospitares.

Análises estatísticas uni e multivariada dos fatores associados ao NMM foram realizadas por meio de regressão logística não condicional, seguindo o modelo hierárquico [23] apresentado na Figura 1. No nível distal, foram incluídas variáveis ​​socioeconômicas e demográficas: idade (12-19 anos , 20-34 anos, ≥35 anos); nível de escolaridade (≤ 7 anos, 8-10 anos, 11-14 anos, 15 anos ou mais de escolaridade); cor da pele auto-referida (branca, preta, parda, amarela, indígena); situação conjugal (vive com o parceiro ou não); paridade (primíparas ou não), e número de cesáreas anteriores (nenhuma, uma, duas ou mais). No nível intermediário, foram incluídas as variáveis ​​relacionadas com a gravidez: assistência pré-natal (pelo menos uma consulta); complicações clínicas ou obstétricas (sim ou não); e número de maternidades procuradas antes da admissão hospitalar (nenhuma, uma, duas ou mais serviços). No nível proximal, a única variável foi o tipo de parto (Cesariana eletiva, Cesariana intraparto, parto vaginal, parto a forceps). O desfecho utilizado foi a incidência de NMM.

Complicações clínicas ou obstétricas foram definidas como condições que constituem possíveis indicações para cesariana e que podem estar potencialmente associadas ao aumento da morbidade materna. Utilizando dados do prontuário hospitalar, foram considerados casos de complicação clínica ou obstétrica as mulheres que apresentaram uma das seguintes condições: distúrbios hipertensivas (hipertensão crônica, pré-eclâmpsia, eclâmpsia e síndrome HELPP), diabetes, placenta prévia, descolamento prematuro da placenta, infecção pelo HIV, e outras infecções maternas no momento da admissão hospitalar.

As cesarianas foram classificadas com base em informações registradas nos prontuários hospitalares. As cesarianas foram classificadas como eletivas se: a) a mulher não apresentou trabalho de parto; ou b) a mulher apresentou trabalho de parto espontâneo ou induzido, mas foi submetida à cesariana com dilatação uterina inferior a 4 cm [24]. Todas as demais cesarianas foram classificados como intraparto, independentemente da duração do trabalho de parto ou da indicação da cesariana .

Casos de NMM foram identificados de acordo com os critérios da OMS [14], usando a informação contida no prontuário hospitalar. Todos os casos identificados foram analisados ​​por dois especialistas independentes, visando detectar possíveis inconsistências decorrentes da qualidade do registro hospitalar ou da extração dos dados. Discordâncias foram resolvidas por consenso.

Estimativas brutas da razão de chances (RC) e respectivos intervalos de confiança (IC 95%) foram obtidas na análise univariada. Na análise multivariada, o primeiro modelo incluiu todas as variáveis ​​distais. Variáveis ​​do primeiro modelo com um nível de significância <0,20 foram incluídas no segundo modelo, juntamente com todas as variáveis ​​do nível intermediário. O terceiro modelo incluiu todas as variáveis ​​do nível distal e intermediário que apresentaram nível de significância de <0,20, junto com as variáveis ​​proximais. Todas as variáveis ​​com nível de significância de <0,05 foram mantidas no modelo multivariado final. Os resultados do modelo multivariado final foram expressos como razão de chance ajustada com seus correspondentes intervalos de confiança (IC 95%).

Para analisar a associação de variáveis ​​socioeconômicas e demográficas com variáveis ​​relacionadas à gravidez, utilizou-se o teste estatístico qui-quadrado para verificar diferenças entre proporções com um nível de significância <0,05.

O desenho amostral complexo foi levado em consideração em todas as análises estatísticas. A ponderação dos dados foi calculada pelo inverso da probabilidade de inclusão de cada puérpera na amostra. Para assegurar que a distribuição das mulheres entrevistadas fosse semelhante à observada entre os nascimentos da população amostrada em 2011, um procedimento de calibração foi utilizada em cada estrato de seleção [22]. Para as regressões logísticas uni e multivariada, as mulheres que se auto-declararm como amarelas ou indígenas foram excluídos da análise, por representarem uma proporção muito pequena da amostra (1,5%). As análises foram realizadas com o programa estatístico IBM SPSS Statistics para Windows, versão 19.0 (IBM Corp., Armonk, NY, EUA).

Este estudo foi aprovado pelo Comitê de Ética em Pesquisa da ENSP / Fiocruz parecer número 92/2010. Todos os cuidados foram adotados para garantir a privacidade e confidencialidade das informações obtidas. O consentimento para a realização da pesquisa foi obtido antes da realização da entrevista com utilização do termo de consentimento livre e esclarecido.

**Resultados**

A média de idade das 23.894 puérperas entrevistadas foi de 25,7 anos, com mediana de 25 anos, estando 19,1% na faixa etária entre 12 e 19 anos. Metade das participantes frequentaram a escola por até 10 anos e a maioria auto-declarou sua cor da pele como parda (56,1%), com amarelas e indígenas correspondendo a 1,1% e 0,4% da amostra, respectivamente. Mais de 80% das mulheres viviam com um parceiro, 46,9% eram primíparas, e 16,2% tinham cesarianas prévias. Cerca de 99% das puérperas tiveram pelo menos uma consulta de pré-natal, com uma média de 7,17 e mediana de 7 consultas; 16,2% procurou uma ou mais maternidades antes da admissão para o parto; e 19,2% apresentaram pelo menos uma complicação clínica ou obstétrica durante a gravidez, parto ou pós-parto. Quase metade das mulheres teve um parto vaginal, enquanto 43,7% tiveram uma cesariana eletiva, 8,2% uma cesariana intraparto e 1,5% um parto vaginal operatório a fórceps (Tabela 1).

A incidência de NMM foi de 10,2 por 1.000 nascidos vivos (IC 95%: 7,5-13,7 por 1000). Na análise univariada, foi observada maior incidência de NMM entre as mulheres com idade igual ou superior a 35 anos, em mulheres com duas ou mais cesarianas prévias, em mulheres que procuraram duas ou mais maternidades antes da admissão para o parto, em mulheres que tiveram complicações clínicas ou obstétricas durante gravidez, e em mulheres que tiveram uma cesariana eletiva ou um parto a fórceps. Mulheres sem assistência pré-natal apresentaram incidência de NMM de 27,97 por 1.000 nascidos vivos, com RC de 2,90, mas significância estatística limítrofe (IC 95%: 0,94-8,92; valor de p = 0,064). A paridade também apresentou significância limítrofe (valor de p = 0,061), mas com uma RC menor (1,3) e um intervalo de confiança estreito (0,99-1,79). Não foram observadas diferenças significativas em relação ao nível de escolaridade, cor da pele auto-referida ou situação conjugal (Tabela 2).

Os resultados da análise ajustada são apresentados na Tabela 3. No primeiro modelo (Modelo 1) a idade da mulher, nível de escolaridade, paridade e número de cesarianas prévias apresentaram um nível de significância <0,20 e foram incluídos no segundo modelo (Modelo 2). No segundo modelo, nível de escolaridade, paridade, cesarianas prévias e todas as variáveis ​​intermediárias apresentaram um nível de significância <0,20 e foram incluídos no terceiro modelo (Modelo 3). No terceiro modelo, apenas as variáveis ​​intermediárias (assistência pré-natal, busca de dois ou mais serviços, complicações clínicas ou obstétricas) e variáveis ​​proximais (tipo de parto) apresentaram um nível de significância <0,05 e foram mantidas no modelo final (Modelo final).

Mulheres que não receberam assistência pré-natal apresentaram uma RC 4,65 maior de NMM (IC 95%: 1,51-14,31) do que mulheres que tiveram pelo menos uma consulta de pré-natal. Mulheres que relataram a busca de duas ou mais maternidades antes da admissão para o parto apresentaram uma RC de incidência de NMM quatro vezes maior do que aquelas que foram admitidas no primeiro serviço de assistência ao parto (RC: 4,49; IC 95%: 2,12-9,52). Mulheres com complicações clínicas ou obstétricas apresentaram a maior RC de NMM na análise não ajustada e uma RC de 9,29 (IC 95%: 6.69-12.90) após ajuste para as demais variáveis incluídas no modelo. Mulheres com cesariana eletiva apresentaram uma RC de 2,54 (IC 95%: 1,67-3,88) após ajuste para demais variáveis, enquanto que as mulheres com um parto a fórceps apresentaram a maior RC para incidência de NMM (RC: 9,37; IC 95%: 4,01 -21,91).

A Tabela 4 mostra a associação de características sociais e demográficas maternas com assistência pré-natal, acesso a serviços de atenção ao parto e complicações clínicas ou obstétricas. Mulheres com 35 anos ou mais apresentaram maior proporção de complicações clínicas ou obstétricas e de cesarianas eletivas, enquanto que mulheres com menos de 20 anos relataram em maior proporção a procura de mais de um serviço para atenção ao parto. Não foram observadas diferenças relacionadas à idade da mãe para assistência pré-natal. Mulheres com menos de 8 anos de estudo relataram menor realização de assistência pré-natal. Observou-se um gradiente de realização de cesarianas eletivas segundo número de anos de estudo: quanto maior o nível de escolaridade, maior a proporção de cesarianas eletivas. Inversamente, a proporção de mulheres que relataram a busca de um ou mais serviços de assistência ao parto antes da internação aumentou à medida que o número de anos de escolaridade diminuiu. As mulheres que se auto-declararam como pretas ou pardas tiveram menos assistência pré-natal, relataram a busca de mais de um serviço de internação para o parto e tiveram menor proporção de cesarianas eletivas; enquanto mulheres que se auto-declararam pretas apresentaram mais complicações clínicas e obstétricas. Observou-se um padrão semelhante entre as mulheres sem parceiro: menor acesso ao pré-natal e à assistência ao parto e menor proporção de cesarianas eletivas. Mulheres com cesáreas anteriores tiveram mais complicações durante a gravidez, mas apresentaram melhor acesso aos serviços de atenção ao parto e quase três vezes mais cesarianas eletivas do que mulheres sem cesarianas anteriores.

**Discussão**

Este estudo estimou uma incidência de NMM de 10,2 por 1.000 nascidos vivos durante a internação hospitalar para a assistência ao parto. Taxas semelhantes foram encontradas em estudos realizados por Souza et al. [25], [26] e Morse et al. [27], que também utilizaram critérios da OMS para definição de casos de NMM. Galvão et al. [28], em um estudo realizado em duas maternidades públicas no estado de Sergipe/Brasil, relataram uma taxa menor de NMM, de 4,7/1.000 nascidos vivos. A comparação dos resultados obtidos neste estudo com outros estudos [29] é limitada, já que foram utilizados os critérios para definição de NMM da OMS, enquanto outros estudos utilizaram critérios diferentes, adotados anteriormente, com diferentes definições de caso de NMM [30].

Neste estudo, a incidência de NMM mostrou associação com ausência de realização de pré-natal (PN), busca de duas ou mais maternidades para admissão para assistência ao parto, complicações clínicas ou obstétricas, e tipo de parto (cesariana electiva e fórceps).

Estudos têm demonstrado a efetividade de diferentes práticas utilizadas rotineiramente na assistência PN para evitar a morbidade e mortalidade [31] materna e perinatal [32]. Estudos observacionais também demonstraram os benefícios dessa assistência, correlacionando maior número de consultas com resultados mais favoráveis ​​[33] - [37], embora não haja consenso sobre o número ideal de consultas de pré-natal [38], [39]. Neste estudo, as mulheres foram classificadas como tendo recebido assistência pré-natal se relataram ter recebido pelo menos uma consulta durante a gravidez. Embora a realização de apenas uma consulta não seja a prática recomendada, muitos procedimentos podem ser realizados em uma única consulta, tais como a testagem e o tratamento para infecção pela sífilis e pelo HIV; o diagnóstico de complicações clínicas, como síndromes hipertensivas; o aconselhamento sobre fatores de risco; o encaminhamento para serviços de gravidez de alto risco; e a vinculação à maternidade de referência para atenção ao parto. Neste estudo, mais de 70% das mulheres apresentaram o número apropriado de consultas, considerando o mínimo de seis consultas recomendado pelo Ministério da Saúde Brasileiro para uma gestação a termo. A ausência de assistência PN mostrou-se associada à menor número de anos de estudo, à cor da pele preta ou parda, à viver sem companheiro e à história de partos anteriores. Resultados da pesquisa "Nascer no Brasil" publicados anteriormente [9] demonstraram associação da ausência de assistência PN com moradia na região Norte, a menos desenvolvida do país; com resultados perinatais negativos em gestação anterior; e com insatisfação com a gravidez atual. Os motivos mais frequentes para não realização do PN foram barreiras de acesso e problemas pessoais (43,2% e 40,6%, respectivamente). Esses resultados sugerem que, no Brasil, a ausência de pelo menos uma consulta pré-natal, em um contexto de cobertura de assistência pré-natal quase universal, está associada a vulnerabilidades sociais e barreiras de acesso a serviços de saúde, aumentando o risco de resultados adversos.

A busca por duas ou mais maternidades para admissão hospitalar para assistência ao parto acarreta demora no acesso a cuidados adequados, com efeitos adversos como complicações e morte [40], [41]. Pacagnella et al. [42], em um estudo transversal nacional multicêntrico em 27 hospitais brasileiros, mostrou que a ocorrência de qualquer demora está associada com o aumento da gravidade dos resultados maternos: 52% condições potencialmente fatais, 68,4% NMM, e 84,1% óbitos maternos. Numa avaliação geral, qualquer tipo de atraso foi observada em 53,8% dos casos e 34,6% das demoras estavam relacionadas à acessibilidade aos serviços de saúde. Embora as leis brasileiras [43] e protocolos [44] regulamentem a vinculação de mulheres grávidas à maternidade de referência para assistência ao parto, neste estudo 16% das mulheres relataram a busca de um ou mais serviços para assistência ao parto. Este resultado revela uma falta de integração entre o atendimento pré-natal e ao parto. A busca de um ou mais serviços mostrou-se associada à menor idade, à menos anos de escolaridade, à cor da pele preta ou parda, à história de partos anteriores e à não viver com um parceiro. Essas são características das mulheres que utilizam os serviços públicos de atenção ao parto. Mulheres com melhores condições sociais e econômicas, atendidas em serviços particulares, geralmente não tem que procurar mais de um serviço, já que normalmente o mesmo profissional presta a assistência pré-natal e ao parto.
Complicações clínicas e obstétricas são as principais causas de mortalidade e morbidade materna grave, e reforçam a importância de cuidados adequados para as mulheres com gravidez de alto risco. Os critérios de complicações clínicas ou obstétricas adotadas neste estudo incluíram patologias que podem resultar em condições potencialmente fatais, como doenças hipertensivas, hemorragias, sepse ou infecção sistêmica grave [45]. A infecção pelo HIV foi incluída por ter afetado o declínio da mortalidade materna em alguns países [46] e ser uma indicação frequente de cesariana.

Tanto cesariana eletiva quanto parto a forceps apresentaram associação com NMM. Cesarianas têm sido descritas como causa de morte materna [12], [13] e NMM [13] e estão associadas a risco aumentado de transfusão sanguínea [11], [12], complicações hemorrágicas [47], infecções [47], [48], histerectomias [11], [12], [48], [49], admissão em unidade de terapia intensiva [11], hospitalização por mais de 7 dias [11], e tratamento com antibióticos após o parto [11]. Neste estudo, após o ajuste para complicações na gravidez, variáveis ​​sociodemográficas e assistência pré-natal, a cesariana eletiva mais do que duplicou as chances de NMM. Resultados semelhantes foram descritos por Villar et al. [11], em estudo em 410 unidades de saúde em 24 regiões de oito países latino-americanos selecionados aleatoriamente. Mulheres com parto vaginal a forceps tiveram as maiores chances de NMM após ajuste para as outras variáveis. No entanto, apenas 1,5% dos partos foram a forceps e muito poucos casos de NMM podem realmente ser atribuídos ao uso de fórceps. Embora a cesariana eletiva apresente uma razão de chances mais baixa do que o parto a fórceps, seu efeito sobre o NMM é maior porque as cesarianas eletivas correspondem a mais de 40% dos partos realizados no Brasil.

A proporção de cesarianas tem aumentado no Brasil desde meados dos anos 1990 e desde 2009 é o principal tipo de parto no país [50]. As maiores proporções de cesariana tem sido observadas em mulheres com idade mais elevada, em mulheres com maior escolaridade, em mulheres primíparas, em mulheres que recebem assistência pré-natal em serviços privados, e em mulheres residentes nas regiões Sul, Sudeste e Centro-Oeste do país [51], sendo determinada, em muitos casos, por fatores não clínicos [52] - [55]. A proporção de cesarianas nos serviços privados é usualmente de 80% a 90%, sendo 80% das cesarianas realizadas antes do início do trabalho de parto [21]. Deve-se ressaltar que estudo recente [56] corroborou a afirmação de que, a nível populacional, uma taxa de cesariana superior a 10 a 15% dificilmente pode ser justificada do ponto de vista clínico. A OMS estima que mais de 1 milhão de cesarianas desnecessárias são realizados no Brasil a cada ano [57].

A história de cesariana prévia não apresentou associação com NMM após o ajuste para as demais variáveis do modelo, sendo os resultados de outros estudos sobre esse tema controversos [23, 58]. Neste estudo, mais de 70% das mulheres grávidas com uma cesariana anterior foram submetidos a uma nova cesariana. Esses resultados sugerem que o risco de NMM associado à cesariana prévia é mitigado pela cesariana atual e que, na gravidez atual, é o tipo de parto que responde pela maior parte do risco associado ao NMM, por ser uma variável mais próxima do desfecho.
A idade tem sido relatada como um fator de risco para NMM [59]. Neste estudo, as mulheres com idade igual ou superior a 35 anos, bem como aquelas que se auto-declararam pretas, eram mais propensas a ter complicações clínicas ou obstétricas; no entanto, após o ajuste para as demais variáveis, as características socioeconômicas e demográficas não apresentaram associação com NMM. Uma possível explicação para este achado, da menor importância das características sociais e econômicas para o desfecho NMM, pode ser demonstrada pela análise de dois grupos distintos. O primeiro, composto por mulheres que se auto-declararam brancas, com 8 ou mais anos de estudo, com maior acesso a serviços de pré-natal e mais frequentemente admitidas no primeiro serviço de atenção ao parto, mas com maior proporção de cesarianas eletivas. E o segundo, composto por mulheres que se auto-declararam como pretas ou pardas, com menos de 8 anos de estudo, com menor acesso a assistência pré-natal e relato de busca de mais de um serviço para admissão para o parto, mas que apresentaram menor proporção de cesarianas eletivas. Esses resultados sugerem que a alta proporção de cesarianas eletivas está atenuando os benefícios para a saúde materna advindos da assistência pré-natal e do maior acesso a serviços de atenção ao parto - tipicamente observados em mulheres com melhores condições socioeconômicas - igualando o risco de NMM entre essas mulheres e aquelas vivendo em piores condições socioeconômicas que, teoricamente, teriam um risco mais elevado de resultados adversos.

Souza et al. [25] relataram um efeito protetor de baixa escolaridade materna para a ocorrência de NMM na pesquisa global sobre saúde materna e perinatal da OMS realizada em 2005. Estudos tem relatado aumento da morbidade materna associados à cesariana na América Latina [10], [11], onde mulheres com baixa escolaridade são menos submetidas à cesariana. Os autores sugerem que o aumento das taxas de cesariana pode estar relacionada à morbidade materna iatrogênica e mortes maternas [25].
Resultados semelhantes foram descritos para a mortalidade neonatal na região Sul do Brasil [60]. Um aumento da taxa de prematuridade, observada durante o período 1982-2004, parece ter resultado, em grande parte, do aumento de cesarianas e induções. Embora os cuidados neonatais tenham melhorado, e as taxas de mortalidade neonatal específicas segundo idade gestacional tenham caído, a taxa de mortalidade neonatal manteve-se estável desde 1990, provavelmente por causa do aumento dos nascimentos prematuros. Os autores concluem que o uso excessivo de intervenções durante a gravidez e o parto pode ter atenuado os ganhos resultantes da melhoria da saúde materna e da maior sobrevida dos recém-natos.

Este estudo foi realizado em instituições onde mais de 500 partos ocorrem a cada ano. É provável que as mulheres grávidas que tiveram partos domiciliares, planejados ou não, ou que tiveram parto hospitalar em hospitais de menor porte tenham riscos diferentes para NMM. No entanto, dado que mais de 99% dos partos realizados no Brasil são hospitalares, e aproximadamente 80% ocorrem em hospitais maiores [20], mudanças significativas para os resultados apresentados não são esperados.

Abortamentos não foram incluídos neste estudo, o que pode ter afetado a estimativa da incidência de NMM, já que abortos são uma causa conhecida de NMM e morte. Um dos objetivos deste estudo foi determinar a associação de NMM com o tipo de parto; a exclusão de abortos não afeta esta análise. É possível, no entanto, que outros factores estejam associados ao NMM em casos de aborto.

Os casos de NMM foram identificados por meio de informações disponíveis no prontuário hospitalar. É possível que falhas no registro tenham resultado em subestimação dos casos de NMM. Como esse seria um erro de classificação não diferencial em relação aos fatores estudados, espera-se que tenha ocorrido uma atenuação da magnitude das associações observadas.

Finalmente, os critérios da OMS adotados para a classificação dos casos de NMM pode dificultar a comparação com os resultados de outros estudos que utilizaram critérios diferentes. É provável, no entanto, que a análise dos fatores associados aos casos de NMM não seja afetada pelos critérios utilizados, embora um estudo tenha sugerido que quando critérios de manejo são usados ​​isoladamente [61], há uma tendência de incluir casos menos graves, o que pode limitar a comparação dos fatores de risco.

**Conclusão**

Os resultados deste estudo demonstram que a ausência de assistência pré-natal, complicações durante a gravidez, busca por dois ou mais serviços de assistência ao parto, e tipo de parto (cesariana electiva e fórceps) estão associados a casos de NMM durante a hospitalização para assistência ao parto no Brasil. Duas estratégias parecem necessárias para reduzir a taxa de NMM. Para as mulheres com maior vulnerabilidade social, é necessário ampliar o acesso à assistência pré-natal e aos serviços de atenção ao parto visando o diagnóstico precoce da gestação, a prestação de cuidados pré-natais adequados e a vinculação da gestante à maternidade para assistência ao parto. Para as mulheres com melhores condições sociais e econômicas, atendidas em grande parte em serviços privados - onde as taxas de cesariana alcançam 90% dos nascimentos - estratégias para reduzir a taxa de cesariana são essenciais.

**Lista de abreviações**

RMM Razão de Mortalidade Materna

NMM Near Miss Materno

OMS Organização Mundial de Saúde

PN Pré-natal

**Conflito de interesses**

Os autores declaram não ter conflito de interesses.

**Contribuições dos autores**

RMSMD fez contribuições substanciais para a concepção e desenho do estudo, coleta e análise de dados, e elaborou o manuscrito; MABD e AOCS participaram da análise dos dados e na revisão

**Agradecimentos**

Este trabalho foi financiado pelo Conselho Nacional de Desenvolvimento Científico e Tecnológico (CNPq); pela Escola Nacional de Saúde Pública, Fundação Oswaldo Cruz (INOVA Projeto); e pela Agência de Financiamento à Pesquisa do Estado do Rio de Janeiro (FAPERJ).

**Referências**

1. Szwarcwald CL, Escalante JJC, Rabello Neto DL, Souza Junior PRB, Victora CG. Estimação da razão de mortalidade materna no Brasil, 2008-2011. Cad Saude Publica. 2014; 30:S71-S83.

2. [Paim J](http://www.ncbi.nlm.nih.gov/pubmed/?term=Paim%20J%5BAuthor%5D&cauthor=true&cauthor_uid=21561655), [Travassos C](http://www.ncbi.nlm.nih.gov/pubmed/?term=Travassos%20C%5BAuthor%5D&cauthor=true&cauthor_uid=21561655), [Almeida C](http://www.ncbi.nlm.nih.gov/pubmed/?term=Almeida%20C%5BAuthor%5D&cauthor=true&cauthor_uid=21561655), [Bahia L](http://www.ncbi.nlm.nih.gov/pubmed/?term=Bahia%20L%5BAuthor%5D&cauthor=true&cauthor_uid=21561655), [Macinko J](http://www.ncbi.nlm.nih.gov/pubmed/?term=Macinko%20J%5BAuthor%5D&cauthor=true&cauthor_uid=21561655). The Brazilian health system: history, advances, and challenges. [Lancet.](http://www.ncbi.nlm.nih.gov/pubmed/21561655) 2011; 377(9779):1778-97.

3. Victora CG, Aquino EML, Leal MC, Monteiro CA, Barros FC, Szwarcwald CL. Maternal and child health in Brazil: progress and challenges. Lancet. 2011; 377 (9780):1863-76.

4. Victora CG, Matijasevich A, Silveira MF, Santos IS, Barros AJD, Barros FC. Socio-economic and ethnic group inequities in antenatal care quality in the public and private sector in Brazil. Health Policy Plan. 2010; 25:253-61.

5. Cesar JA, Mano OS, Carlotto K, Gonzalez-Chica DA, Mendoza-Sassi RA. Público *versus* privado: avaliando a assistência à gestação e ao parto no extremo sul do Brasil Rev. Bras. Saude Matern. Infant. 2011; 11: 257-263.

6. Andreucci CB, Cecatti JG. Desempenho de indicadores de processo do Programa de Humanização do Pré-natal e Nascimento no Brasil: uma revisão sistemática. Cad Saude Publica. 2011; 27: 1053-1064.

7. [Domingues RM](http://www.ncbi.nlm.nih.gov/pubmed?term=Domingues%20RM%5BAuthor%5D&cauthor=true&cauthor_uid=24896600)SM, [Leal M do C](http://www.ncbi.nlm.nih.gov/pubmed?term=Leal%20Mdo%20C%5BAuthor%5D&cauthor=true&cauthor_uid=24896600), [Hartz ZM](http://www.ncbi.nlm.nih.gov/pubmed?term=Hartz%20ZM%5BAuthor%5D&cauthor=true&cauthor_uid=24896600), [Dias MA](http://www.ncbi.nlm.nih.gov/pubmed?term=Dias%20MA%5BAuthor%5D&cauthor=true&cauthor_uid=24896600), [Vettore MV](http://www.ncbi.nlm.nih.gov/pubmed?term=Vettore%20MV%5BAuthor%5D&cauthor=true&cauthor_uid=24896600). Access to and utilization of prenatal care services in the Unified Health System of the city of Rio de Janeiro, Brazil. [Rev Bras Epidemiol.](http://www.ncbi.nlm.nih.gov/pubmed/24896600) 2013; 16: 953-65.

8. [Bernardes AC](http://www.ncbi.nlm.nih.gov/pubmed?term=Bernardes%20AC%5BAuthor%5D&cauthor=true&cauthor_uid=25108701), [da Silva RA](http://www.ncbi.nlm.nih.gov/pubmed?term=da%20Silva%20RA%5BAuthor%5D&cauthor=true&cauthor_uid=25108701), [Coimbra LC](http://www.ncbi.nlm.nih.gov/pubmed?term=Coimbra%20LC%5BAuthor%5D&cauthor=true&cauthor_uid=25108701), [Alves MT](http://www.ncbi.nlm.nih.gov/pubmed?term=Alves%20MT%5BAuthor%5D&cauthor=true&cauthor_uid=25108701), [Queiroz RC](http://www.ncbi.nlm.nih.gov/pubmed?term=Queiroz%20RC%5BAuthor%5D&cauthor=true&cauthor_uid=25108701), [Batista RF](http://www.ncbi.nlm.nih.gov/pubmed?term=Batista%20RF%5BAuthor%5D&cauthor=true&cauthor_uid=25108701) et al. Inadequate prenatal care utilization and associated factors in Sao Luis, Brazil. [BMC Pregnancy Childbirth.](http://www.ncbi.nlm.nih.gov/pubmed/25108701) 2014; 14: 266.

9. Viellas EF, Domingues RMSM, Domingues RMSM , Dias MAB, da Gama SGN, Theme Filha MM et al. Assistência pré-natal no Brasil. Cad Saude Publica. 2014; 30: S85-S100.

10. Villar J, Valladares E, Wojdyla D, Zavaleta N, Carroli G, Velazco A et al. Caesarian delivery rates and pregnancy outcomes: the 2005 WHO global survey on maternal and perinatal health in Latin America. Lancet. 2006; 367:1819-29.

11. [Villar J](http://www.ncbi.nlm.nih.gov/pubmed/?term=Villar%20J%5BAuthor%5D&cauthor=true&cauthor_uid=17977819), [Carroli G](http://www.ncbi.nlm.nih.gov/pubmed/?term=Carroli%20G%5BAuthor%5D&cauthor=true&cauthor_uid=17977819), [Zavaleta N](http://www.ncbi.nlm.nih.gov/pubmed/?term=Zavaleta%20N%5BAuthor%5D&cauthor=true&cauthor_uid=17977819), [Donner A](http://www.ncbi.nlm.nih.gov/pubmed/?term=Donner%20A%5BAuthor%5D&cauthor=true&cauthor_uid=17977819), [Wojdyla D](http://www.ncbi.nlm.nih.gov/pubmed/?term=Wojdyla%20D%5BAuthor%5D&cauthor=true&cauthor_uid=17977819), [Faundes A](http://www.ncbi.nlm.nih.gov/pubmed/?term=Faundes%20A%5BAuthor%5D&cauthor=true&cauthor_uid=17977819) et al. [World Health Organization 2005 Global Survey on Maternal and Perinatal Health Research Group](http://www.ncbi.nlm.nih.gov/pubmed/?term=World%20Health%20Organization%202005%20Global%20Survey%20on%20Maternal%20and%20Perinatal%20Health%20Research%20Group%5BCorporate%20Author%5D). Maternal and neonatal individual risks and benefits associated with caesarean delivery: multicentre prospective study. [BMJ.](http://www.ncbi.nlm.nih.gov/pubmed/17977819) 2007; 335(7628):1025.

12. Souza JP, Gülmezoglu AM, Lumbiganon P, Laopaiboon M, Carroli G, Fawole B et al. Caesarean section without medical indications is associated with an increased risk of adverse short-term maternal outcomes: The 2004–2008 WHO Global Survey on Maternal and Perinatal Health. BMC Med. 2010; 8:71.

13. [Litorp H](http://www.ncbi.nlm.nih.gov/pubmed/?term=Litorp%20H%5BAuthor%5D&cauthor=true&cauthor_uid=25056517), [Kidanto HL](http://www.ncbi.nlm.nih.gov/pubmed/?term=Kidanto%20HL%5BAuthor%5D&cauthor=true&cauthor_uid=25056517), [Rööst M](http://www.ncbi.nlm.nih.gov/pubmed/?term=R%C3%B6%C3%B6st%20M%5BAuthor%5D&cauthor=true&cauthor_uid=25056517), [Abeid M](http://www.ncbi.nlm.nih.gov/pubmed/?term=Abeid%20M%5BAuthor%5D&cauthor=true&cauthor_uid=25056517), [Nyström L](http://www.ncbi.nlm.nih.gov/pubmed/?term=Nystr%C3%B6m%20L%5BAuthor%5D&cauthor=true&cauthor_uid=25056517), [Essén B](http://www.ncbi.nlm.nih.gov/pubmed/?term=Ess%C3%A9n%20B%5BAuthor%5D&cauthor=true&cauthor_uid=25056517). Maternal near-miss and death and their association with caesarean section complications: a cross-sectional study at a university hospital and a regional hospital in Tanzania. [BMC Pregnancy Childbirth.](http://www.ncbi.nlm.nih.gov/pubmed/?term=lithorp+%26+maternnal+%26deaths) 2014; 14:244.

14. Say L, Souza JP, Pattinson RC. Maternal near miss – towards a standard tool for monitoring quality of maternal health care. Best Pract Res Clin Obstet Gynaecol. 2009; 23:287-96.

15. [Tunçalp Ö](http://www.ncbi.nlm.nih.gov/pubmed/?term=Tun%C3%A7alp%20%C3%96%5BAuthor%5D&cauthor=true&cauthor_uid=25236642), [Souza JP](http://www.ncbi.nlm.nih.gov/pubmed/?term=Souza%20JP%5BAuthor%5D&cauthor=true&cauthor_uid=25236642). Maternal near-miss audits to improve quality of care. [BJOG.](http://www.ncbi.nlm.nih.gov/pubmed/25236642) 2014; 121:102-4.

16. Pattinson RC, Hall M. Near misses: a useful adjunct to maternal death enquiries. Br Med Bull. 2003; 67:231-43.

17. Pattinson R. Near miss audit in obstetrics. Best Pract Res Clin Obstet Gynaecol. 2009; 23:285-6.

18. Souza JP, Cecatti JG, Haddad SM, Parpinelli MA, Costa ML, Katz L et al. The WHO maternal near-miss approach and the maternal severity index model (MSI): tools for assessing the management of severe maternal morbidity. PLoS One. 2012; 7:e44129.

19. [Dias MA](http://www.ncbi.nlm.nih.gov/pubmed/?term=Dias%20MA%5BAuthor%5D&cauthor=true&cauthor_uid=25167176), [Domingues RM](http://www.ncbi.nlm.nih.gov/pubmed/?term=Domingues%20RM%5BAuthor%5D&cauthor=true&cauthor_uid=25167176), [Schilithz AO](http://www.ncbi.nlm.nih.gov/pubmed/?term=Schilithz%20AO%5BAuthor%5D&cauthor=true&cauthor_uid=25167176), [Nakamura-Pereira M](http://www.ncbi.nlm.nih.gov/pubmed/?term=Nakamura-Pereira%20M%5BAuthor%5D&cauthor=true&cauthor_uid=25167176), [Diniz CS](http://www.ncbi.nlm.nih.gov/pubmed/?term=Diniz%20CS%5BAuthor%5D&cauthor=true&cauthor_uid=25167176), [Brum IR](http://www.ncbi.nlm.nih.gov/pubmed/?term=Brum%20IR%5BAuthor%5D&cauthor=true&cauthor_uid=25167176)et al. Incidence of maternal near miss in hospital childbirth and postpartum: data from the Birth in Brazil study. [Cad Saude Publica.](http://www.ncbi.nlm.nih.gov/pubmed/?term=Incidence+of+maternal+near+miss+in+hospital+childbirth+and+postpartum%3A+data+from+the+Birth+in+Brazil+study) 2014; 30:S1-12.

20. do Carmo Leal M, da Silva AA, Dias MA, Gama SG, Rattner D, Moreira ME et al. Birth in Brazil: national survey into labour and birth. Reprod Health. 2012; 9:15.

21. Domingues RMSM, Dias MAB, Nakamura-Pereira M, Torres JA, d’Orsi E, Pereira APE, et al. Processo de decisão pelo tipo de parto no Brasil: da preferência inicial das mulheres à via de parto final. Cad Saude Publica. 2014; 30:S101-16.

22. Vasconcellos MTL, Silva PLN, Pereira APE, Schilithz AOC, Souza Junior PRB, Szwarcwald CL. Sampling design for the Birth in Brazil: National Survey into Labor and Birth. Cad Saude Publica. 2014; 30: S49-S58.

23. [Victora CG](http://www.ncbi.nlm.nih.gov/pubmed/?term=Victora%20CG%5BAuthor%5D&cauthor=true&cauthor_uid=9126524), [Huttly SR](http://www.ncbi.nlm.nih.gov/pubmed/?term=Huttly%20SR%5BAuthor%5D&cauthor=true&cauthor_uid=9126524), [Fuchs SC](http://www.ncbi.nlm.nih.gov/pubmed/?term=Fuchs%20SC%5BAuthor%5D&cauthor=true&cauthor_uid=9126524), [Olinto MT](http://www.ncbi.nlm.nih.gov/pubmed/?term=Olinto%20MT%5BAuthor%5D&cauthor=true&cauthor_uid=9126524). The role of conceptual frameworks in epidemiological analysis: a hierarchical approach. [Int J Epidemiol.](http://www.ncbi.nlm.nih.gov/pubmed/9126524) 1997; 26:224-7.

24. Safe prevention of the primary cesarean delivery. Obstetric Care Consensus No. 1. American College of Obstetricians and Gynecologists. Obstet Gynecol 2014;123:693–711.

25. [Souza JP](http://www.ncbi.nlm.nih.gov/pubmed/?term=Souza%20JP%5BAuthor%5D&cauthor=true&cauthor_uid=20428368), [Cecatti JG](http://www.ncbi.nlm.nih.gov/pubmed/?term=Cecatti%20JG%5BAuthor%5D&cauthor=true&cauthor_uid=20428368), [Faundes A](http://www.ncbi.nlm.nih.gov/pubmed/?term=Faundes%20A%5BAuthor%5D&cauthor=true&cauthor_uid=20428368), [Morais SS](http://www.ncbi.nlm.nih.gov/pubmed/?term=Morais%20SS%5BAuthor%5D&cauthor=true&cauthor_uid=20428368), [Villar J](http://www.ncbi.nlm.nih.gov/pubmed/?term=Villar%20J%5BAuthor%5D&cauthor=true&cauthor_uid=20428368), [Carroli G](http://www.ncbi.nlm.nih.gov/pubmed/?term=Carroli%20G%5BAuthor%5D&cauthor=true&cauthor_uid=20428368) et al. [World Health Organization 2005 Global Survey on Maternal and Perinatal Health Research Group](http://www.ncbi.nlm.nih.gov/pubmed/?term=World%20Health%20Organization%202005%20Global%20Survey%20on%20Maternal%20and%20Perinatal%20Health%20Research%20Group%5BCorporate%20Author%5D). Maternal near miss and maternal death in the World Health Organization’s 2005 global survey on maternal and perinatal health. [Bull World Health Organ.](http://www.ncbi.nlm.nih.gov/pubmed/?term=Maternal+near+miss+and+maternal+death+in+the+World+Health+Organization%E2%80%99s+2005+global+survey+on+maternal+and+perinatal+health) 2010; 88:113-9.

26. Souza JP, Gülmezoglu AM, Vogel J, Carroli G, Lumbiganon P, Qureshi Z et al. Moving beyond essential interventions for reduction of maternal mortality (the WHO Multicountry Survey on Maternal and Newborn Health): a cross-sectional study. Lancet. 2013; 381:1747-55.

27. Morse ML, Fonseca SC, Gottgtroy CL, Waldmann CS, Gueller E. Severe maternal morbidity and near misses in a regional reference hospital. Rev Bras Epidemiol. 2011; 14:310-22.

28. Galvão LP, Alvim-Pereira F, de Mendonça CM, Menezes FE, Góis KA, Ribeiro RF Jr et al. [The prevalence of severe maternal morbidity and near miss and associated factors in Sergipe, Northeast Brazil.](http://www.ncbi.nlm.nih.gov/pubmed/24433516) BMC Pregnancy Childbirth. 2014; 14:25.

29. [Souza JP](http://www.ncbi.nlm.nih.gov/pubmed/?term=Souza%20JP%5BAuthor%5D&cauthor=true&cauthor_uid=16501738), [Cecatti JG](http://www.ncbi.nlm.nih.gov/pubmed/?term=Cecatti%20JG%5BAuthor%5D&cauthor=true&cauthor_uid=16501738), [Parpinelli MA](http://www.ncbi.nlm.nih.gov/pubmed/?term=Parpinelli%20MA%5BAuthor%5D&cauthor=true&cauthor_uid=16501738), [de Sousa MH](http://www.ncbi.nlm.nih.gov/pubmed/?term=de%20Sousa%20MH%5BAuthor%5D&cauthor=true&cauthor_uid=16501738), [Serruya SJ](http://www.ncbi.nlm.nih.gov/pubmed/?term=Serruya%20SJ%5BAuthor%5D&cauthor=true&cauthor_uid=16501738). Systematic review of near miss maternal morbidity. [Cad Saude Publica.](http://www.ncbi.nlm.nih.gov/pubmed/16501738) 2006; 22:255-64.

30. [Lobato G](http://www.ncbi.nlm.nih.gov/pubmed/?term=Lobato%20G%5BAuthor%5D&cauthor=true&cauthor_uid=23182071), [Nakamura-Pereira M](http://www.ncbi.nlm.nih.gov/pubmed/?term=Nakamura-Pereira%20M%5BAuthor%5D&cauthor=true&cauthor_uid=23182071), [Mendes-Silva W](http://www.ncbi.nlm.nih.gov/pubmed/?term=Mendes-Silva%20W%5BAuthor%5D&cauthor=true&cauthor_uid=23182071), [Dias MA](http://www.ncbi.nlm.nih.gov/pubmed/?term=Dias%20MA%5BAuthor%5D&cauthor=true&cauthor_uid=23182071), [Reichenheim ME](http://www.ncbi.nlm.nih.gov/pubmed/?term=Reichenheim%20ME%5BAuthor%5D&cauthor=true&cauthor_uid=23182071). Comparing different diagnostic approaches to severe maternal morbidity and near-miss: a pilot study in a Brazilian tertiary hospital. [Eur J Obstet Gynecol Reprod Biol.](http://www.ncbi.nlm.nih.gov/pubmed/23182071) 2013; 167:24-8.

31. Carroli G, Rooney C, Villar J. How effective is antenatal care in preventing maternal mortality and serious morbidity? An overview of the evidence. Paediatr Perinat Epidemiol. 2001; 15:S1-42.

32. Villar J, Carroli G, Khan-Neelofur D, Piaggio G, Gülmezoglu M. Patterns of routine antenatal care for low-risk pregnancy. Cochrane Database Syst Rev. 2001; (4):CD000934.

33. Silveira SD, Santos IS. Adequação do pré-natal e peso ao nascer: uma revisão sistemática. Cad Saude Publica. 2004; 20:1160-8.

34. Leal MC, Gama SGN, Ratto KMN, Cunha CB. Uso do índice de Kotelchuck modificado na avaliação da assistência pré-natal e sua relação com as características maternas e o peso do recém-nascido no Município do Rio de Janeiro. Cad Saude Publica. 2004; 20:63-72.

35. Schoeps D, Almeida MF, Alencar GP, França Jr. I, Novaes HMD, Siqueira AAF et al. Fatores de risco para mortalidade neonatal precoce. Rev Saude Publica. 2007; 41:1013-22.

36. Wehby GL, Murray JC, Castilla EE, Lopez-Camelo JS, Ohsfeldt RL. Prenatal care effectiveness and utilization in Brazil. Health Policy Plan. 2009; 24:175-88.

37. Debiec KE, Paul KJ, Mitchell CM, Hitti JE. Inadequate prenatal care and risk of preterm delivery among adolescents: a retrospective study over 10 years. Am J Obstet Gynecol. 2010; 203:122.e1-6.

38. Villar J, Ba’aqeel H, Piaggio G, Lumbiganon P, Miguel Belizán J, Farnot U et al. WHO Antenatal Care Trial Research Group. WHO antenatal care randomised trial for the evaluation of a new model of routine antenatal care. Lancet. 2001; 357(9268):1551-1564.

39. [Hofmeyr GJ](http://www.ncbi.nlm.nih.gov/pubmed?term=Hofmeyr%20GJ%5BAuthor%5D&cauthor=true&cauthor_uid=23577750), [Hodnett ED](http://www.ncbi.nlm.nih.gov/pubmed?term=Hodnett%20ED%5BAuthor%5D&cauthor=true&cauthor_uid=23577750). Antenatal care packages with reduced visits and perinatal mortality: a secondary analysis of the WHO antenatal care trial - Commentary: routine antenatal visits for healthy pregnant women do make a difference. [Reprod Health.](http://www.ncbi.nlm.nih.gov/pubmed/?term=Hofmeyr+and+Hodnett+Reproductive+Health+2013%2C+10%3A20) 2013; 10:20.

40. Thaddeus S, Maine D. Too far to walk: maternal mortality in context. Soc Sci Med. 1994; 38:1091-110.

41. Thorsen VC, Sundby J, Malata A. Piecing together the maternal death puzzle through narratives: the three delays model revisited. PLoS One. 2012; 7:e52090.

42. [Pacagnella RC](http://www.ncbi.nlm.nih.gov/pubmed/?term=Pacagnella%20RC%5BAuthor%5D&cauthor=true&cauthor_uid=24886330), [Cecatti JG](http://www.ncbi.nlm.nih.gov/pubmed/?term=Cecatti%20JG%5BAuthor%5D&cauthor=true&cauthor_uid=24886330)1, [Parpinelli MA](http://www.ncbi.nlm.nih.gov/pubmed/?term=Parpinelli%20MA%5BAuthor%5D&cauthor=true&cauthor_uid=24886330), [Sousa MH](http://www.ncbi.nlm.nih.gov/pubmed/?term=Sousa%20MH%5BAuthor%5D&cauthor=true&cauthor_uid=24886330), [Haddad SM](http://www.ncbi.nlm.nih.gov/pubmed/?term=Haddad%20SM%5BAuthor%5D&cauthor=true&cauthor_uid=24886330), [Costa ML](http://www.ncbi.nlm.nih.gov/pubmed/?term=Costa%20ML%5BAuthor%5D&cauthor=true&cauthor_uid=24886330) et al. [Brazilian Network for the Surveillance of Severe Maternal Morbidity study group](http://www.ncbi.nlm.nih.gov/pubmed/?term=Brazilian%20Network%20for%20the%20Surveillance%20of%20Severe%20Maternal%20Morbidity%20study%20group%5BCorporate%20Author%5D). Delays in receiving obstetric care and poor maternal outcomes: results from a national multicentre cross-sectional study. [BMC Pregnancy Childbirth.](http://www.ncbi.nlm.nih.gov/pubmed/24886330) 2014; 14:159.

43. Brasil. Lei no 11.634, de 27 de dezembro de 2007. Dispõe sobre o direito da gestante ao conhecimento e a vinculação à maternidade onde receberá assistência no âmbito do SUS. Diário Oficial da União 2007; 28 dez. http://www.planalto.gov.br/ccivil_03/_Ato2007-2010/2007/Lei/L11634.htm. Accessed 15 April 2015.

44. Brasil. Ministério da Saúde. Portaria consolidada Rede Cegonha. <http://bvsms.saude.gov.br/bvs/saudelegis/gm/2011/prt1459_24_06_2011.html>.

Accessed 26 February 2015.

45. World Health Organization. Evaluating the quality of care for severe pregnancy complications: the WHO near-miss approach for maternal health. Geneva: World Health Organization; 2011.

46. [Hogan MC](http://www.ncbi.nlm.nih.gov/pubmed?term=%22Hogan%20MC%22%5BAuthor%5D), [Foreman KJ](http://www.ncbi.nlm.nih.gov/pubmed?term=%22Foreman%20KJ%22%5BAuthor%5D), [Naghavi M](http://www.ncbi.nlm.nih.gov/pubmed?term=%22Naghavi%20M%22%5BAuthor%5D), [Ahn SY](http://www.ncbi.nlm.nih.gov/pubmed?term=%22Ahn%20SY%22%5BAuthor%5D), [Wang M](http://www.ncbi.nlm.nih.gov/pubmed?term=%22Wang%20M%22%5BAuthor%5D), [Makela SM](http://www.ncbi.nlm.nih.gov/pubmed?term=%22Makela%20SM%22%5BAuthor%5D) et al. Maternal mortality for 181 countries, 1980-2008: a systematic analysis of progress towards Millennium Development Goal 5. [Lancet.](http://lancet./) 2010; 375:1609-23.

47. Karlström A, Lindgren H, Hildingsson I. Maternal and infant outcome after caesarean section without recorded medical indication: findings from a Swedish case–control study. BJOG. 2013; 120:479-486.

48. Liu S, Liston RM, Joseph KS, Heaman M, Sauve R, Kramer MS; Maternal Health Study Group of the Canadian Perinatal Surveillance System. [Maternal mortality and severe morbidity associated with low-risk planned cesarean delivery versus planned vaginal delivery at term.](http://www.ncbi.nlm.nih.gov/pubmed/17296957) CMAJ. 2007; 176:455-60.

49. Kamilya G, Seal SL, Mukherji J, Bhattacharyya SK, Hazra A. Maternal mortality and cesarean delivery: an analytical observational study. J Obstet Gynaecol Res. 2010; 36:248-253.

50. Brasil. Ministério da Saúde. DataSus.

<http://tabnet.datasus.gov.br/cgi/tabcgi.exe?sinasc/cnv/nvuf.def>. Accessed 23 February 2015.

51. Rebelo F, Rocha CMM, Cortes TR, Dutra CL, Kac G. High cesarean prevalence in a national population-based study in Brazil: the role of private practice. Acta Obstet Gynecol Scand. 2010; 89:903-8.

52. Dias MAB, Domingues RMSM, Pereira AP, Fonseca SC, Gama SGN, Theme-Filha MM et al. Trajetória das mulheres na definição pelo parto cesáreo: estudo de caso em duas unidades do sistema de saúde suplementar do estado do Rio de Janeiro. Cienc Saude Coletiva. 2008; 13:1521-34.

53. Hopkins K. Are Brazilian women really choosing to deliver by cesarean? Soc Sci Med. 2000; 51:725-40.

54. Behague DP, Victora CG, Barros FC. Consumer demand for caesarean sections in Brazil: informed decision making, patient choice or social inequality? A population based birth cohort study linking ethnographic and epidemiological methods. BMJ. 2002; 324:942-5.

55. Barros AJD, Santos IS, Matijasevich A, Domingues MR, Silveira M, Barros FC et al. Patterns of deliveries in a Brazilian birth cohort: almost universal cesarean sections for the better-off. Rev Saude Publica. 2011; 45:635-43

56. [Ye J](http://www.ncbi.nlm.nih.gov/pubmed/?term=Ye%20J%5BAuthor%5D&cauthor=true&cauthor_uid=24720614), [Betrán AP](http://www.ncbi.nlm.nih.gov/pubmed/?term=Betr%C3%A1n%20AP%5BAuthor%5D&cauthor=true&cauthor_uid=24720614), [Guerrero Vela M](http://www.ncbi.nlm.nih.gov/pubmed/?term=Guerrero%20Vela%20M%5BAuthor%5D&cauthor=true&cauthor_uid=24720614), [Souza JP](http://www.ncbi.nlm.nih.gov/pubmed/?term=Souza%20JP%5BAuthor%5D&cauthor=true&cauthor_uid=24720614), [Zhang J](http://www.ncbi.nlm.nih.gov/pubmed/?term=Zhang%20J%5BAuthor%5D&cauthor=true&cauthor_uid=24720614). Searching for the optimal rate of medically necessary cesarean delivery. [Birth.](http://www.ncbi.nlm.nih.gov/pubmed/24720614) 2014; 41:237-44.

57. Gibbons L, Belizán JM, Lauer JA, Betrán AP, Merialdi M & Althabe F. The global numbers and costs of additionally needed and unnecessary caesarean sections performed per year: overuse as a barrier to universal coverage. World Health Report Background Paper N^o^ 30. Geneva: World Health Organization; 2010.

58. [Litorp H](http://www.ncbi.nlm.nih.gov/pubmed/?term=Litorp%20H%5BAuthor%5D&cauthor=true&cauthor_uid=26868073), [Rööst M](http://www.ncbi.nlm.nih.gov/pubmed/?term=R%C3%B6%C3%B6st%20M%5BAuthor%5D&cauthor=true&cauthor_uid=26868073), [Kidanto HL](http://www.ncbi.nlm.nih.gov/pubmed/?term=Kidanto%20HL%5BAuthor%5D&cauthor=true&cauthor_uid=26868073), [Nyström L](http://www.ncbi.nlm.nih.gov/pubmed/?term=Nystr%C3%B6m%20L%5BAuthor%5D&cauthor=true&cauthor_uid=26868073), [Essén B](http://www.ncbi.nlm.nih.gov/pubmed/?term=Ess%C3%A9n%20B%5BAuthor%5D&cauthor=true&cauthor_uid=26868073). The effects of previous cesarean deliveries on severe maternal and adverse perinatal outcomes at a university hospital in Tanzania. [Int J Gynaecol Obstet.](http://www.ncbi.nlm.nih.gov/pubmed/?term=the+effects+of+previous+cesarean+deliveries+on+severe+maternal+and+adverse+perinatal+outcomes+at+a+university+hospital+in+Tanzania.+IJGO%2C+2016) 2016. pii: S0020-7292(16)00006-0. [Epub ahead of print].

59. [Oliveira FC Jr](http://www.ncbi.nlm.nih.gov/pubmed/?term=Oliveira%20FC%20Jr%5BAuthor%5D&cauthor=true&cauthor_uid=24555831), [Surita FG](http://www.ncbi.nlm.nih.gov/pubmed/?term=Surita%20FG%5BAuthor%5D&cauthor=true&cauthor_uid=24555831), [Pinto E Silva JL](http://www.ncbi.nlm.nih.gov/pubmed/?term=Pinto%20E%20Silva%20JL%5BAuthor%5D&cauthor=true&cauthor_uid=24555831), [Cecatti JG](http://www.ncbi.nlm.nih.gov/pubmed/?term=Cecatti%20JG%5BAuthor%5D&cauthor=true&cauthor_uid=24555831), [Parpinelli MA](http://www.ncbi.nlm.nih.gov/pubmed/?term=Parpinelli%20MA%5BAuthor%5D&cauthor=true&cauthor_uid=24555831), [Haddad SM](http://www.ncbi.nlm.nih.gov/pubmed/?term=Haddad%20SM%5BAuthor%5D&cauthor=true&cauthor_uid=24555831) et al. [Brazilian Network for Surveillance of Severe Maternal Morbidity Study Group](http://www.ncbi.nlm.nih.gov/pubmed/?term=Brazilian%20Network%20for%20Surveillance%20of%20Severe%20Maternal%20Morbidity%20Study%20Group%5BCorporate%20Author%5D). Severe maternal morbidity and maternal near miss in the extremes of reproductive age: results from a national cross- sectional multicenter study. [BMC Pregnancy Childbirth.](http://www.ncbi.nlm.nih.gov/pubmed/24555831) 2014; 14:77.

60. Barros FC, Victora CG, Barros AJ, Santos IS. The challenge of reducing neonatal mortality in middle-income countries: findings from three Brazilian birth cohorts in 1982, 1993, and 2004. Lancet. 2005; 365:847-54.

61. Cecatti JG, Souza JP, Oliveira Neto AF, Parpinelli MA, Sousa MH, Say L et al. Pre-validation of the WHO organ dysfunction based criteria for identification of maternal near miss. Reprod Health. 2011; 8:22.

**Tabela 1. Distribuição proporcional das puérperas (n=23.894) incluídas no estudo de hospitalização para o parto segundo características maternas, Brasil, 2011–2012**

| **Característica** | **n** | **%** |
| --- | --- | --- |
| **Idade materna (anos)** |  |  |
| 12**–**19 | 4,571 | 19.1 |
| 20**–**34 | 16,807 | 70.4 |
| 35 ou mais | 2,509 | 10.5 |
| **Escolaridade (anos de estudo)** |  |  |
| 15 ou mais | 2,107 | 8.9 |
| 11**–**14 | 9,263 | 39.0 |
| 8**–**10 | 6,086 | 25.6 |
| 0**–**7 | 6,322 | 26.6 |
| **Cor da pele** |  |  |
| Branca | 8,077 | 33.8 |
| Parda | 13,404 | 56.1 |
| Preta | 2,051 | 8.6 |
| Amarela | 257 | 1.1 |
| Indígena | 99 | 0.4 |
| **Situação conjugal** |  |  |
| Com parceiro | 19,440 | 81.4 |
| Sem parceiro | 4,432 | 18.6 |
| **Número de partos anteriores** |  |  |
| 0 | 11,208 | 46.9 |
| 1 | 7,014 | 29.4 |
| 2-3 | 4,501 | 18.8 |
| 4 ou mais | 1,171 | 4.9 |
| **Cesariana anterior** |  |  |
| 0 | 18,779 | 78.6 |
| 1 | 3,905 | 16.3 |
| 2 ou mais | 1,211 | 5.1 |
| **Número de consultas de pré-natal** |  |  |
| 0 | 286 | 1.2 |
| 1-3 | 2,123 | 9.1 |
| 4-5 | 4,110 | 17.6 |
| 6 ou mais | 16,898 | 72.1 |
| **Número de serviços antes da admissão para o parto** |  |  |
| 0 | 20,005 | 83.8 |
| 1 | 3,302 | 13.8 |
| 2 ou mais | 569 | 2.4 |
| **Complicações clínicas ou obstétricas** |  |  |
| Não | 19,264 | 80.6 |
| Sim | 4,630 | 19.4 |
| **Tipo de complicação clínica ou obstétrica ^1^** |  |  |
| Distúrbios hipertensivos | 2,656 | 11.1 |
| Diabetes mellitus | 1,968 | 8.2 |
| Descolamento premature da placenta | 310 | 1.3 |
| Placenta prévia | 116 | 0.5 |
| Infecção materna | 83 | 0.3 |
| Infecção pelo HIV | 96 | 0.4 |
| **Tipo de parto** |  |  |
| Vaginal | 11,152 | 46.7 |
| Cesariana eletiva | 10,436 | 43.7 |
| Cesariana intraparto | 1,959 | 8.2 |
| Forceps/ | 347 | 1.5 |

Valores totais diferem por causa de valores sem informação ^1^ Apenas mulheres com complicação clínica ou obstétrica (n=4.630).

**Tabela 2. Incidência de *Near Miss* Materno (NMM), razão de chance (RC), Intervalo de confiança 95% e valor de p em puérperas incluídas no estudo (n=23.894) segundo características maternas, Brasil, 2011–2012**

| **Característica** | **Incidência de NMM ^1^** | **RC^2^** | **IC 95% ^3^** | **Valor de**  **p** |
| --- | --- | --- | --- | --- |
| **Idade materna (anos)** |  |  |  |  |
| 12**–**19 | 10.06 | 1.07 | 0,72–1.60 |  |
| 20**–**34 | 9.40 | 1 | 1 | 0.081 |
| 35 ou mais | 15.54 | 1.65 | 1.07–2.55 |  |
| **Escolaridade (anos de estudo)** |  |  |  |  |
| 15 ou mais | 6.17 | 1 |  |  |
| 11**–**14 | 10.04 | 1.61 | 0.75–3.45 |  |
| 8**–**10 | 11.18 | 1.81 | 0.84–3.92 | 0.494 |
| 0**–**7 | 10.91 | 1.75 | 0.72–4.22 |  |
| **Cor da pele^4^** |  |  |  |  |
| Branca | 9.29 | 1 |  |  |
| Parda | 10.67 | 1.16 | 0.79–1.70 | 0.746 |
| Preta | 9.75 | 1.08 | 0.45–2.56 |  |
| **Situação conjugal** |  |  |  |  |
| Com parceiro | 10.02 | 1 |  |  |
| Sem parceiro | 10.83 | 1.08 | 0.77–1.52 | 0.651 |
| **Primípara** |  |  |  |  |
| Não | 8.82 | 1 |  |  |
| Sim | 11.68 | 1.33 | 0.99–1.79 | 0.061 |
| **Cesariana prévia** |  |  |  |  |
| 0 | 9.58 | 1 |  |  |
| 1 | 10.84 | 1.13 | 0.75–1.72 |  |
| 2 ou mais | 17.05 | 1.79 | 1.09-2.97 | 0.035 |
| **Pré-natal** |  |  |  |  |
| Sim | 9.87 | 1 |  |  |
| Não | 27.97 | 2.90 | 0.94–8.92 | 0.064 |
| **Número de serviços antes da admissão para o parto** |  |  |  |  |
| 0 | 8.85 | 1 |  |  |
| 1 | 13.93 | 1.59 | 0.89–2.83 |  |
| 2 ou mais | 35.15 | 4.10 | 1.97–8.52 | 0.001 |
| **Complicações clínicas**  **ou obstétricas^5^** |  |  |  |  |
| Não | 3.63 | 1 |  |  |
| Sim | 37.15 | 10.55 | 7.61–14.63 | <0.001 |
| **Tipo de parto** |  |  |  |  |
| Vaginal | 4.39 | 1 |  |  |
| Cesariana eletiva | 16.00 | 3.70 | 2.47–5.55 |  |
| Cesariana Intraparto | 6.64 | 1.48 | 0.76–2.88 |  |
| Forceps | 40.35 | 9.64 | 4.24–21.91 | <0.001 |
| **TOTAL** | 10.16 | -- | 7.14–13.18 | -- |

Valores totais diferem por causa de valores sem informação.^1^ Incidência de *near miss* materno por 1.000 nascidos vivos. ^2^ RC = razão de chance; ^3^ IC = interval de confiança; ^4^ mulheres que se auto-declararam amarelas ou indígenas foram excluídas desta análise; ^5^ mulheres que apresentaram um dos seguintes critérios foram classificadas como tendo complicação clínica ou obstétrica: distúrbios hipertensivos, diabetes, placenta prévia, descolamento prematuro de placenta, infecção pelo HIV e outras infecções maternas.

**Tabela 3. Regressão logística multivariada das características maternas associadas à incidência *near miss* materno durante a hospitalização para o parto (n=23.894), Brasil, 2011–2012**

| **Características** | **Modelo 1^1^** | | | **Modelo 2^2^** | | | **Modelo 3^3^** | | | **Modelo Final^4^** | | |
| --- | --- | --- | --- | --- | --- | --- | --- | --- | --- | --- | --- | --- |
|  | RC^5^ | 95% CI^6^ | P | OR^5^ | IC 95% ^6^ | p | RC^5^ | IC 95% ^6^ | p | RC^5^ | IC 95% ^6^ | p |
| **Idade Materna (anos)** |  |  |  |  |  |  |  |  |  |  |  |  |
| 12**–**19 | 0.71 | 0.48-1.06 |  | 0.96 | 0.60-1.52 |  |  |  |  |  |  |  |
| 20 **–**34 | 1 |  |  | 1 |  |  |  |  |  |  |  |  |
| 35 ou mais | 1.93 | 1.25-2.97 | 0.001 | 1.37 | 0.87-2.17 | 0.358 |  |  |  |  |  |  |
| **Escolaridade**  **(anos de estudo)** |  |  |  |  |  |  |  |  |  |  |  |  |
| 15 ou mais | 1 |  |  | 1 |  |  | 1 |  |  |  |  |  |
| 11**–**­­ 14 | 1.96 | 0.91-4.24 |  | 1.65 | 0.81-3.39 |  | 1.78 | 0.85-3.73 |  |  |  |  |
| 8 **–** 10 | 2.49 | 1.15-5.37 |  | 2.17 | 1.04-4.54 |  | 2.48 | 1.15-5.33 |  |  |  |  |
| 0 **–** 7 | 2.66 | 1.04-6.78 | 0.106 | 2.07 | 0.84-5.08 | 0.185 | 2.51 | 0.99-6.36 | 0.075 |  |  |  |
| **Cor da pele ^7^** |  |  |  |  |  |  |  |  |  |  |  |  |
| Branca | 1 |  |  |  |  |  |  |  |  |  |  |  |
| Parda | 1.15 | 0.76-1.75 |  |  |  |  |  |  |  |  |  |  |
| Preta | 1.05 | 0.43-2.56 | 0.777 |  |  |  |  |  |  |  |  |  |
| **Situação Conjugal** |  |  |  |  |  |  |  |  |  |  |  |  |
| Com parceiro | 1 |  |  |  |  |  |  |  |  |  |  |  |
| Sem parceiro | 0.97 | 0.67-1.42 | 0.893 |  |  |  |  |  |  |  |  |  |
| **Primípara** |  |  |  |  |  |  |  |  |  |  |  |  |
| Não | 1 |  |  | 1 |  |  | 1 |  |  |  |  |  |
| Sim | 2.40 | 1.53-3.77 | <0.001 | 2.02 | 1.26-3.25 | 0.004 | 1.45 | 0.90-2.34 | 0.131 |  |  |  |
| **Cesariana prévia** |  |  |  |  |  |  |  |  |  |  |  |  |
| 0 | 1 |  |  | 1 |  |  | 1 |  |  |  |  |  |
| 1 | 1.89 | 1.06-3.38 |  | 1.61 | 0.91-2.88 |  | 1.07 | 0.62-1.86 |  |  |  |  |
| 2 ou mais | 2.64 | 1.62-4.30 | <0.001 | 2.13 | 1.31-3.45 | 0.003 | 1.30 | 0.74-2.29 | 0.638 |  |  |  |
| **Pré-natal** |  |  |  |  |  |  |  |  |  |  |  |  |
| Sim |  |  |  | 1 |  |  | 1 |  |  | 1 |  |  |
| Não |  |  |  | 4.15 | 1.34-12.81 | 0.014 | 4.44 | 1.37-14.37 | 0.013 | 4.65 | 1,51-14.31 | 0,007 |
| **Número de serviços antes da admissão para o parto** |  |  |  |  |  |  |  |  |  |  |  |  |
| 0 |  |  |  | 1 |  |  | 1 |  |  | 1 |  |  |
| 1 |  |  |  | 1.50 | 0.84-2.68 |  | 1.57 | 0.89-2.75 |  | 1.69 | 0.98-2.92 |  |
| 2 ou mais |  |  |  | 3.88 | 1.84-8.18 | 0.002 | 4.05 | 1.92-8.54 | <0.001 | 4.49 | 2.12-9.52 | <0.001 |
| **Complicações clínicas ou obstétricas^8^** |  |  |  |  |  |  |  |  |  |  |  |  |
| Não |  |  |  | 1 |  |  | 1 |  |  | 1 |  |  |
| Sim |  |  |  | 10.28 | 7.51-14.06 | <0.001 | 9.26 | 6.66-12.87 | <0.001 | 9.29 | 6.69-12.90 | <0.001 |
| **Tipo de parto** |  |  |  |  |  |  |  |  |  |  |  |  |
| Vaginal |  |  |  |  |  |  | 1 |  |  | 1 |  |  |
| Cesariana eletiva |  |  |  |  |  |  | 2.67 | 1.70-4.18 |  | 2.54 | 1.67-3.88 |  |
| Cesariana Intraparto |  |  |  |  |  |  | 1.02 | 0.53-1.94 |  | 1.05 | 0.54-2.03 |  |
| Forceps |  |  |  |  |  |  | 9.08 | 3.92-21.06 | <0.001 | 9.37 | 4.01-21.91 | <0.001 |

^1^ Modelo 1 = análises ajustadas para idade maternal, escolaridade, cor da pele auto declarada, situação conjugal, paridade e número de cesarianas anteriores; ^2^ Modelo 2 = análises ajustadas para idade, escolaridade, paridade, número de cesarianas anteriores, assistência pré-natal, diagnóstico de complicações clínicas ou obstétricas, número de serviços procurados antes da admissão para o parto; ^3^ Modelo 3 = análises ajustadas para escolaridade, paridade, número de cesarianas anteriores, assistência pré-natal, diagnóstico de complicações clínicas ou obstétricas, número de serviços procurados antes da admissão para o parto e tipo de parto; ^4^ Modelo Final = análises ajustadas para assistência pré-natal, diagnóstico de complicações clínicas ou obstétricas, número de serviços procurados antes da admissão para o parto e tipo de parto ^5^RC = razão de chance; ^6^ IC = intervalo de confiança; ^7^Mulheres que se auto-declararam amarelas ou indígenas foram excluídas desta análise; ^8^ mulheres que apresentaram um dos seguintes critérios foram classificadas como tendo complicação clínica ou obstétrica: distúrbios hipertensivos, diabetes, placenta previa, descolamento prematuro de placenta, infecção pelo HIV e outras infecções maternas.

**Tabela 4. Assistência pré-natal, procura por serviços de atenção ao parto, complicações clínicas e obstétricas e cesariana eletiva segundo características maternas, Brasil, 2011–2012**

| **Característica** | **Assistência Pré-Natal** | **Valor de p^1^** | **Procura por um ou mais serviços antes da admissão** | **Valor de p^1^** | **Complicações clínicas ou obstétricas** | **Valor de p^1^** | **Cesariana eletiva** | **Valor de p^1^** |
| --- | --- | --- | --- | --- | --- | --- | --- | --- |
|  |  |  |  |  |  |  |  |  |
| **Idade Materna (anos)** |  |  |  |  |  |  |  |  |
| 12**–**19 | 98.5 |  | 21.3 |  | 12.9 |  | 26.8 |  |
| 20**–**34 | 98.8 |  | 15.4 |  | 19.2 |  | 46.0 |  |
| 35 ou mais | 98.9 | 0.745 | 12.5 | <0.001 | 32.2 | <0.001 | 59.0 | <0.001 |
| **Escolaridade (anos)** |  |  |  |  |  |  |  |  |
| 15 ou mais | 100 |  | 5.9 |  | 20.5 |  | 79.5 |  |
| 11**–**14 | 99.5 |  | 14.0 |  | 20.8 |  | 50.9 |  |
| 8**–**10 | 98.9 |  | 18.5 |  | 17.8 |  | 35.4 |  |
| 0**–**7 | 97.3 | <0.001 | 20.7 | <0.001 | 18.5 | 0.009 | 29.0 | <0.001 |
| **Cor da pele ^2^** |  |  |  |  |  |  |  |  |
| Branca | 99.3 |  | 12.2 |  | 20.3 |  | 53.1 |  |
| Parda | 98.6 |  | 18.1 |  | 18.5 |  | 39.5 |  |
| Preta | 98.5 | 0.009 | 19.6 | <0.001 | 22.4 | 0.004 | 35.0 | <0.001 |
| **Situação Conjugal** |  |  |  |  |  |  |  |  |
| Com parceiro | 99.2 |  | 15.8 |  | 19.8 |  | 45.3 |  |
| Sem parceiro | 97.2 | <0.001 | 18.1 | 0.041 | 17.5 | 0.035 | 36.4 | <0.001 |
| **Primípara** |  |  |  |  |  |  |  |  |
| Não | 98.2 |  | 14.8 |  | 19.8 |  | 41.5 |  |
| Sim | 99.5 | <0.001 | 17.8 | <0.001 | 18.9 | 0.264 | 46.1 | <0.001 |
| **Cesariana prévia** |  |  |  |  |  |  |  |  |
| 0 | 98.8 |  | 17.1 |  | 18.2 |  | 35.0 |  |
| 1 | 99.0 |  | 13.3 |  | 23.2 |  | 71.5 |  |
| 2 ou mais | 98.8 | 0.200 | 11.3 | <0.001 | 25.6 | <0.001 | 89.2 | <0.001 |

^1^ Teste estaístico Qui-Quadrado; ^2^ Mulheres que se auto declararam amarelas ou indígenas foram excluídas desta análise

**Figura 1. Modelo teórioc dos determinantes do *Near Miss* Materno (MNM)**

**Nível distal Nível intermediário Nível proximal Desfecho**

**Características da gestação**

Assistência pré-natal

Complicações clínicas ou obstétricas

Procura por uma ou mais maternidades para admissão para o parto

**Tipo de parto:**

Vaginal

Cesariana eletiva

Cesariana intra-parto

Forceps

**Características demográficas e socioeconômicas**

Escolaridade materna

Idade

Cor da pele

Situação conjugal

Paridade

Cesariana prévia

**NMM**
