# Supplementary material for: Factors associated with maternal near miss in childbirth and the postpartum period: findings from the birth in Brazil National Survey, 2011–2012
Source: Reprod Health. 2016 Oct 17;13(Suppl 3):115. doi: 10.1186/s12978-016-0232-y (PMC5073804; doi:10.1186/s12978-016-0232-y)
Supplement: Additional file 2: — Reviewer reports. (PDF 251 kb) [file 12978_2016_232_MOESM2_ESM.pdf]

Referee's comments to the authors– this sheet WILL be seen by the author(s) and published with the article

Title	Factors associated with maternal near miss in childbirth and the postpartum period: findings from the Birth in Brazil National Survey 2011-2012
Author(s)	Rosa Maria Soares Madeira Domingues, Marcos Augusto Bastos Dias, Arthur Orlando Corrêa Schilithz and Maria do Carmo Leal
Referee's name	Helena Litorp

When assessing the work, please consider the following points, where applicable:

1. Is the question posed by the authors new and well defined?
2. Are the methods appropriate and well described, and are sufficient details provided to replicate the work?
3. Are the data sound and well controlled?
4. Does the manuscript adhere to the relevant standards for reporting and data deposition?
5. Are the discussion and conclusions well balanced and adequately supported by the data?
6. Do the title and abstract accurately convey what has been found?
7. Is the writing acceptable?

Please make your report as constructive and detailed as possible in your comments so that authors have the opportunity to overcome any serious deficiencies that you find and please also divide your comments into the following categories:

- Major Compulsory Revisions (which the author must respond to before a decision on publication can be reached)
- Minor Essential Revisions (such as missing labels on figures, or the wrong use of a term, which the author can be trusted to correct)
- Discretionary Revisions (which are recommendations for improvement but which the author can choose to ignore)

Where possible please supply references to substantiate your comments.

When referring to the manuscript please provide specific page and paragraph citations where appropriate.

General comments:
Generally, I found this paper to be interesting and quite well-written, and it raises important concerns relating to the high use of CS in Brazil.

Major compulsory revisions:
* Method: Generally, I miss some information about the setting: how is maternity care organized in Brazil? Private vs. public clinics? What is the national rate of CS? What about the use of instrumental delivery (vacuum or forceps only? percentage of instrumental delivery on a national level?). As I am not familiar with the Latin American setting, I would want to know more about the "search for maternity services" - how does this work and why do women need to search for delivery services – are many hospitals overcrowded?
* Method: Line 131: From a clinical perspective, I cannot really see the point in exploring the association between "clinical and obstetric complications" and MNM, it seems obvious that there is an association between the severe complications you mention on line 140-141 and MNM (?). As you mention, it might be useful to present this data in order to understand the case mix of the study population and also to interpret your analysis of CSs, but maybe not as a result on its own (like it is presented now).

(continue on the next sheet)

Minor essential revisions:
* Method: How was the distribution between private and public clinics included in the current survey?
* Method: Line 116: Who performed the interviews? Any of the authors? Research assistants? What were the interviewers' relations to the interviewees?
* Method: Line 140-141: What about uterine rupture – was that considered/included as an obstetric complication? If not, why?
* Method: Line 144: Please include a reference for this categorization, I am not sure "failed induction" should be categorized as "elective CSs" (a woman might undergo CS because of "failed induction" although she has been in labour for several hours).
* Results: Line 185: How large proportion of the women had the recommended 4 antenatal care visits? I see that you have mentioned on line 258 in the Discussion that 70% of the women had at least 6 visits, maybe this should be mentioned in the Results?
* Discussion: Line 251-260: You compare your results with other studies, but what is your interpretation of the findings that MNM was associated with absence of antenatal care? What is the reason why some women do not use antenatal care services in this setting? Maybe you can comment here on your findings in Table 4, the sociodemographic characteristics associated with non-use of antenatal care services?
* Discussion: Line 295-304: Some of this information would be good to have in the Method section describing the setting.
* Conclusion: Line 377-379: In my opinion, this line, as well as the reference, suits better in the Discussion section.
* Table 1: Primipara yes or no: As parity > 4 also has been associated with MNM, I think it would be good to present parity with more categories (at least three).
* Table 1: Antenatal care yes or no: As the use of antenatal care services and its association with MNM is one of the findings that you highlight most, I think it would be good to present this variable with more categories (none, 1-4, > 4 or whatever you find suitable) just to give the reader some more idea of how women use antenatal care services in this setting.

Discretionary revisions:
* Abstract: The abbreviation MNM should be mentioned the first time the term is used, that is on line 26 (not line 34).
* Abstract: The first sentence in the conclusion is a bit unclear to me – do you mean that the high rate of elective CSs among women with higher socioeconomic status attenuate the benefit of their better access to prenatal services compared to women with lower socioeconomic status? Or do you mean that there has been improvement over time when it comes to prenatal services etc., but that the effects of these improvements have been attenuated by the high rate of CSs? Please specify.
* Discussion: Line 305-306: We actually just published a paper with the same result; that previous CS was not associated with an increased risk of MNM (Litorp et al. The effects of previous cesarean deliveries on severe maternal and adverse perinatal outcomes at a university hospital in Tanzania. IJGO, 2016).
* Discussion: Line 320 and 323: I would suggest not to use italics.

Referee's comments to the authors– this sheet WILL be seen by the author(s) and published with the article

Title	Factors associated with maternal near miss in childbirth and the postpartum period: findings from the Birth in Brazil National Survey 2011-2012
Author(s)	Rosa Maria Soares Madeira Domingues, Marcos Augusto Bastos Dias, Arthur Orlando Corrêa Schilithz and Maria do Carmo Leal
Referee's name	Rodrigo Vianna

When assessing the work, please consider the following points, where applicable:

1. Is the question posed by the authors new and well defined?
2. Are the methods appropriate and well described, and are sufficient details provided to replicate the work?
3. Are the data sound and well controlled?
4. Does the manuscript adhere to the relevant standards for reporting and data deposition?
5. Are the discussion and conclusions well balanced and adequately supported by the data?
6. Do the title and abstract accurately convey what has been found?
7. Is the writing acceptable?

Please make your report as constructive and detailed as possible in your comments so that authors have the opportunity to overcome any serious deficiencies that you find and please also divide your comments into the following categories:

- Major Compulsory Revisions (which the author must respond to before a decision on publication can be reached)
- Minor Essential Revisions (such as missing labels on figures, or the wrong use of a term, which the author can be trusted to correct)
- Discretionary Revisions (which are recommendations for improvement but which the author can choose to ignore)

Where possible please supply references to substantiate your comments.

When referring to the manuscript please provide specific page and paragraph citations where appropriate.

General comments:
Dear authors, congratulations for your manuscript. It is very relevant, the theme is important to public health and I consider adequate the focus on near miss for two reasons, the methodological one is the possibility to work with greater number of events (and better controlled data) and the social one is to prevent health problems before the death have had occurred.
The background is very clear and any lector, health professional or not, can be sure about the subject importance.
I have no essentials revisions, only some discretionary revisions in order to improve, if the authors agree, the manuscript

Discretionary revisions:
After the well done background, the methods are very clear and objective, but:

a) I had special concern about the antenatal care categories, divided in "*none*" vs "*at least one consultation*". We know, as is cited in the paper discussion part, that more than 70% of the women had six or more antenatal consultation, so, if only 1.2% of the women in the study dataset had none antenatal consultation, these categories could be more flexible, and, for example, divided on none, one to five, six or more.
b) The hierarchical model is adequate and appropriate, based on the figure showed. This methodology is able to find the relationship amongst distal and the event variables, despite the intermediate or proximal levels variables. When these closer variables are included in the model, usually the first one loose significance. Nevertheless I suggest do not exclude the distal variables from the model, in order to adjust the final model.

Continued:

c) After the second consideration, the fourth paragraph, page 7 (line 162), could be rearranged. This analysis explains the relationship amongst the distal variables and the intermediate level. In fact, this analysis showed that these variables are not independent. The associations observed occurs in two different directions, “no antenatal care” and “search for one or more services before admission” are more frequent on younger or poorest mothers and “clinical or obstetric complications” and “elective C-section” are more frequent on older or better socioeconomic conditions mothers. These different directions could reduce the total effect of distal level variables on maternal near miss.

d) Finally, the last methodology suggestion is do not exclude women who self-reported as East Asian or indigenous because they was 1.5% of the sample. No antenatal care was 1.2%, forceps delivery was also 1.5% and these characteristics remain in all analyses.

About the Results:

a) The proportions of women who had had at least one prenatal consultation is too high, this makes the sample also too homogeneous. Not to do prenatal consultation could be a confounding variable, because show no access to health service but only could be happen if the woman is very health.

b) I suggest remove the affirmative starting line 195. It is not make sense borderline statistical significance and this relationship p value (p= 0.064) is greater than parity p-value (0.061) and in this second case the authors said “no significance difference”. So there are two same results, but two different interpretations.

c) The Table 1 shows the percent distribution of study independent variables. The “previous C-section” is described for all mother, included the women who did not had another son. I believe it is different to have already delivery experience with or not C-section than no delivery experience at all. Then, this variable could be rebuilding for women with childbirth experience only or include another category “no previous childbirth experience” (the second is better for the multiple logistic regression).

d) The strength loss of association after adjustment, wrote line 213 is not necessary (and not important).

e) It is impressive the increase of antenatal care OR from the unadjusted analysis to adjusted. It could be discussed later.

About the Discussion:

a) Lines 248 to 250 are only results and could be suppress.

b) The third paragraph on page 13 (line 305) could be different if the change proposed in results, item c), is accepted.

c) In page 14, lines 312 to 328, also could be changed if some different analyses was carry out, like remain the distal significant variables on final model, to stratify the mother according item c) methodology suggestion. This discussion will be impact on Conclusions when future actions are recommended.

d) The paragraph started on line 335 presents one unnecessary analogy about two different actions fighting one against another, and I suggest removing.

e) The third paragraph on the same page (line 349) could be included the Brazilian abortions law description where it is not allowed, but in very specific (and very severe) conditions.

Finally, about the Conclusions:

a) The results do not showed limited access to antenatal care, but a very small part of total women (1.5%) do none antenatal consults and it is a risk factor to maternal near miss. Plan actions involving access to at least one prenatal consultation will be a very low population impact. Aspects related with antenatal quality are not measured in this study.

b) The second recommendation is indeed valid, and very important to avoid the maternity search at the birth time.

c) To reduce elective C-section is imperative and the most important study recommendation, despite the fact of the risk is not so elevated (OR=2.54), it has a high population impact, especially in Brazil where this practice absolutely excessive (43.7% showed in this paper)

d) The 59th citation could be in the discussion. It is better conclusions only with authors words.
